# Economic value and clinical association of a supervised lifestyle-improving program for MASLD

**DOI:** 10.3389/fphar.2025.1708451

**Published:** 2026-01-16

**Authors:** Maurizio Polignano, Antonella Bianco, Davide Guido, Pietro Trisolini, Isabella Franco, Caterina Bonfiglio, Gianluigi Giannelli

**Affiliations:** 1 Clinical Trial Unit - National Institute of Gastroenterology - I.R.C.C.S “Saverio de Bellis”, Castellana Grotte, Italy; 2 Laboratory of Movement & Wellness - National Institute of Gastroenterology - I.R.C.C.S “Saverio de Bellis”, Castellana Grotte, Italy; 3 UOS Data Science, National Institute of Gastroenterology - I.R.C.C.S “Saverio de Bellis”, Castellana Grotte, Italy; 4 Hospital Pharmacy, National Institute of Gastroenterology - I.R.C.C.S “Saverio de Bellis”, Castellana Grotte, Italy; 5 Scientific Direction, National Institute of Gastroenterology – I.R.C.C.S “Saverio de Bellis”, Castellana Grotte, Italy

**Keywords:** metabolic dysfunction-associated steatotic liver disease, lifestyle program, supervised exercise, cost–utility analysis, quality of life

## Abstract

**Background:**

Metabolic dysfunction-associated steatotic liver disease (MASLD) is both common and, in some cases, a progressive condition. Emerging pharmacological options have shown promise in select patient sub-groups (e.g., resmetirom for MASH with fibrosis; GLP-1 receptor agonists for obesity/diabetes with metabolic benefits), but structured lifestyle programs remain foundational in routine care.

**Objective:**

This study evaluates the cost–utility analysis of a multidisciplinary, kinesiology-supervised lifestyle-improving program for patients with MASLD, supported by clinical evidence.

**Methods:**

We analyzed 27 adults with MASLD, a cohort established from an initial group of 43 subjects, who participated in a structured program of supervised exercise and dietary counseling. Health-related quality of life (SF-36 mapped to EQ-5D) and associated clinical markers, including hepatic steatosis (ultrasound), blood pressure, and serum aminotransferases, were evaluated at baseline and after the program. A cost–utility analysis was conducted from the healthcare system’s perspective, estimating the incremental cost-effectiveness ratios (ICERs and €/QALY) with deterministic and probabilistic sensitivity analyses. Pharmaceutical expenditures and projected disease progression costs were also explored using administrative data and literature-based models.

**Results:**

Health-related quality of life improved after the program, with a quality-adjusted life year (QALY) gain of 0.081 (95% CI: 0.001–0.161). The base-case ICER was €17,778/QALY. The probability of cost-effectiveness was 71% at €25,000/QALY, 84% at €30,000/QALY, and 95% at €40,000/QALY. Ultrasound steatosis showed a distributional shift toward lower grades with an unchanged median (Wilcoxon p = 0.007). Systolic/diastolic blood pressure decreased by −5.6/−3.7 mmHg (p = 0.05 and p = 0.03), and AST/ALT declined (both p < 0.01). At the 2-year follow-up, 55.6% of patients reported maintaining regular physical activity. Outpatient pharmaceutical expenditures showed a decline from €74 to €50 per patient/year between 2018 and 2021, with reduced variability across patients. However, this trend did not reach statistical significance in mixed-effects analyses (p = 0.06).

**Conclusion:**

In this pre–post observational study, the supervised program was associated with favorable cost–utility outcomes and distributional improvements in selected clinical markers. These findings support the program’s potential value in routine care and warrant confirmation in controlled studies.

**Clinical Trial Registration:**

https://clinicaltrials.gov/expert-search?term, identifier NCT06026293.

## 1 Introduction

Metabolic dysfunction-associated steatotic liver disease (MASLD) is a major global health issue and is driven by obesity and sedentary behavior, with significant clinical and economic burdens (Girish and John, 2025). Beyond hepatic fat accumulation, MASLD is characterized by systemic metabolic dysregulation and is strongly associated with increased risks of type 2 diabetes, cardiovascular disease, chronic kidney disease, and hepatocellular carcinoma. These extrahepatic consequences contribute more to morbidity and mortality than progression to cirrhosis itself, underscoring MASLD as a multisystem disorder with wide-ranging health and economic consequences (Younossi et al., 2023).

The recent shift in terminology from nonalcoholic fatty liver disease (NAFLD) to MASLD highlights the central role of metabolic dysfunction in the disease definition and research focus (Mantovani et al., 2021; Mantovani et al., 2022). Lifestyle modification remains the recommended first-line approach. Although a therapeutic paradigm shift is emerging for MASH with advanced fibrosis, supported by the recent approvals of pharmacological agents such as Rezdiffra (resmetirom), a once-daily oral thyroid hormone receptor-β (THR-β) agonist that improves hepatic fat metabolism to reduce steatosis, inflammation, and fibrosis (Harrison et al., 2024), and Wegovy (semaglutide), a glucagon-like peptide-1 (GLP-1) receptor agonist that targets both liver disease and its underlying metabolic drivers, including obesity and insulin resistance (Sanyal et al., 2025, p. 3), no drug has yet been broadly approved for MASLD.

Structured programs that combine supervised exercise with dietary counseling can improve hepatic and extrahepatic outcomes through enhanced lipid oxidation, improved insulin sensitivity, and attenuation of low-grade inflammation. However, long-term maintenance remains challenging, and high-quality, real-world evidence of sustained clinical effects and economic value is limited (Eslam et al., 2020; Rinella et al., 2023).

This study evaluates a multidisciplinary, kinesiology-supervised lifestyle program for MASLD implemented in routine care. We performed a cost–utility analysis, recording changes in health-related quality of life (HRQoL) (SF-36 mapped to EQ-5D), hepatic steatosis, blood pressure, and aminotransferase levels. Our aim is to provide evidence relevant to decision-making regarding the clinical and economic sustainability and value of the program in a clinical setting.

## 2 Materials and methods

This single-center, cohort study was conducted at the I. R.C.C.S. “Saverio de Bellis.” Eligible participants were adults (≥18 years) with ultrasound-confirmed MASLD and no contraindications to exercise or concomitant chronic liver disease. Between 2018 and 2020, 58 participants were enrolled in a multidisciplinary lifestyle program consisting of supervised exercise and dietary counseling. The program was interrupted in early 2020 due to the COVID-19 pandemic and was never fully completed. At recall in 2023, 43 individuals attended the follow-up evaluation; of these individuals, 27 who had completed at least two follow-up visits other than the baseline visit, corresponding to a minimum of three valid assessments, were included in the final analysis.

The program lasted 12 months and included thrice-weekly sessions of combined aerobic and resistance training delivered in a hospital-affiliated gym facility under the continuous supervision of kinesiology-trained staff and with medical oversight to ensure safety and adherence. Nutritional counseling was provided by a qualified dietitian and was tailored to individual needs.

HRQoL markers (from questionnaires) and clinical evaluations, including anthropometry, blood sampling, and fitness testing (Franco et al., 2019; Franco et al., 2020; Bianco et al., 2023) were recorded at baseline and every 2 months. Liver ultrasounds and standard biochemical panels were repeated at each visit to allow for standardized longitudinal monitoring. Comprehensive details on eligibility criteria, assessment tools, and scheduling are reported in the Supplementary Material (S1). Pharmacological treatment profiles at baseline (year 2018) were analyzed using regional dispensing data extracted from the cohort under observation. Drug classes were categorized according to the Anatomical Therapeutic Chemical (ATC) classification system, and each patient was assigned to one or more therapeutic groups based on the medications dispensed during enrollment. Drug classes with fewer than three dispensed packages were excluded to avoid noise from occasional or short-term prescriptions.

The therapeutic categories were then mapped to the corresponding clinical conditions and grouped into system organ classes (SOC) according to the Medical Dictionary for Regulatory Activities (MedDRA). This mapping enabled the identification of major comorbidities that are potentially associated with MASLD, including hypertension, dyslipidemia, type 2 diabetes, thyroid dysfunction, gout, osteoporosis, and bacterial infections. Each patient could contribute to more than one disease category when concurrent treatments were present. Descriptive analyses were performed to estimate the prevalence of each pharmacological condition and its relative contribution to the overall comorbidity burden within the cohort.

### 2.1 Cost-effectiveness evaluation

#### 2.1.1 Mapping SF-36 to EQ-5D utility

HRQoL was measured at each assessment using the 36-item short-form health survey (SF-36) (Ware and Sherbourne, 1992; Apolone and Mosconi, 1998), which includes eight subscales: physical functioning, role physical, bodily pain, general health, vitality, social functioning, role emotional, and mental health. To allow for health utility and quality-adjusted life year (QALY) estimation, we mapped SF-36 scores onto EQ-5D utilities using the validated regression algorithm developed by Ara and Wailoo (2011). This mapping is recommended and widely used in cost-effectiveness research when direct EQ-5D data are unavailable.

The utility value for each observation was calculated as follows:

EQ−5D utility=0.03256+0.00370 x Phisical Functioning+0.00111×Social functioning−0.00024×Role Physical+0.00024×Role Emotional+0.00256×Mental Health−0.00063×Vitality+0.00286×Bodily Pain+0.00052×General Health,

where all subscale values are the respective SF-36 domain scores, each normalized to a 0–100 scale. The mapping was applied to all patient records and time points.

Hence, a statistical approach using a linear mixed-effects model was applied to evaluate the QALY gains (i.e., utility) in relation to time and accounting for intra-subject variability.

#### 2.1.2 Costs

Program costs were itemized by component (Table 1), and included gym membership, professional supervision, project management, and insurance. The monthly cost was €120 per patient, amounting to €1,440 annually. All costs were adjusted to 2024 EUR using the harmonized index of consumer prices (HICP) for the Euro area (base year = 2015), as published monthly by Eurostat (Eurostat, 2024).

**TABLE 1:** Breakdown of the direct monthly costs for the supervised exercise program per patient.

Cost component	Monthly cost (€)
Gym membership	30
Expert supervision	60
Project management	20
Insurance policy	10
Total (monthly)	**120**
Total (annual)	**1,440**

Bold values indicate total parameter values used in the main analysis.

#### 2.1.3 Economic evaluation

Incremental cost-effectiveness ratios (ICERs, €/QALY) were calculated, versus a “no-program” scenario (C = 0, E = 0), by dividing the mean per-patient cost of the program by the mean QALY gain observed.

$$\text{ICER}=\frac{C_{1}-C_{0}}{E_{1}-E_{0}}.$$

Here, C_1_ is the average cost for the program group.
C_0_ is the average cost at baseline (no intervention).
E_1_ is the average effect (e.g., QALYs) in the program group.
E_0_ is the average effect at baseline (no intervention).

In addition, the results were expressed using the net monetary benefit (NMB) framework, which is defined as follows:

$$\text{NMB}=\left(QALY_{gain}\times WTP\right)-\text{Cost}.$$

Here, QALY_gain_ is the incremental QALY gained.
WTP is the willingness-to-pay threshold (€/QALY).
Cost is the incremental cost of the program versus the control.

Here, WTP denotes the willingness-to-pay threshold per QALY. The NMB was calculated at €25,000, €30,000, and €40,000 per QALY, in line with the commonly adopted thresholds in Italy and Europe. This is consistent with the international Health Technology Assessment guidance from the NICE, the WHO, and the EUnetHTA (Tan-Torres Edejer et al., 2003; National Institute for Health and Care Excellence NICE, 2013; European Network for Health Technology Assessment EUnetHTA, 2015).

#### 2.1.4 Sensitivity analyses

Deterministic one-way sensitivity analysis (OWSA) (Briggs et al., 2012) was performed by varying key parameters (program cost and QALY gain) across pre-specified ranges while retaining others constant at the base-case value. Results were expressed as differences (Δ) from the base case and visualized using tornado plots.

Probabilistic sensitivity analysis (PSA) (Fenwick et al., 2001; Briggs et al., 2006) was conducted with 10,000 Monte Carlo simulations. QALY gains were sampled from a log-normal distribution, and costs were sampled from a gamma distribution; a Gaussian copula (ρ = 0.25) (Bai et al., 2018) was applied to induce positive correlations between costs and QALY draws. Simulations yielding QALY <0.01 were excluded as implausible. The probability of cost-effectiveness was summarized through cost-effectiveness acceptability curves (CEACs), defined as Pr [NMB(WTP) > 0] across a range of thresholds (€20,000–40,000/QALY).

Health economic analyses (ICER, NMB, one-way sensitivity analysis, and probabilistic sensitivity analysis with standard distributions for costs, utilities, and transition probabilities) were carried out in Python (v3.12.11) using customized, reproducible scripts.

The codebase included parameter validation, random seed control, and modular routines for generating tornado plots, CE planes, CEACs, and structured Excel outputs. Reporting followed the Consolidated Health Economic Evaluation Reporting Standards (CHEERS 2022) to ensure methodological transparency and comparability with the international literature (Husereau et al., 2022) (Supplementary Material S2).

### 2.2 Clinical association

Assessments were scheduled at baseline and at 2, 4, 6, and 8 months (i.e., every 2 months), with ultrasounds, blood tests, and questionnaires repeated at each visit.

#### 2.2.1 Measurements

Hepatic steatosis was assessed by liver ultrasound (LUS) using an Esaote MyLab A70 XVG device equipped with a 5 MHz convex probe. Evaluations were conducted at baseline and throughout the study period. A semi-quantitative scoring system was utilized to assess hepatic fat accumulation based on three sonographic parameters: (1) contrast between hepatic and renal parenchymal echogenicity, (2) attenuation of the ultrasound beam with depth penetration, and (3) clarity of the intrahepatic vascular structures, particularly the portal and hepatic veins.

Based on this scoring system, hepatic steatosis was categorized as follows: absent (score 0), mild (score 1–2), moderate (score 3–4), or severe (score 5–6). During each examination, additional ultrasound parameters, such as liver size, contour, and echotexture, were also evaluated. To maximize repeatability and reduce measurement variability, all assessments were performed by a single, highly experienced operator using the same instrument with fixed acquisition settings throughout the study, thereby eliminating inter-operator and inter-instrument variabilities.

Blood pressure was measured following the European Society of Hypertension (Williams et al., 2018) guidelines using calibrated automated sphygmomanometers; three consecutive seated readings were obtained, and the mean value was used for analysis.

Laboratory assessments included serum lipids, glucose, liver enzymes (ALT, AST, and GGT), insulin, and HbA1c, which were collected at baseline and every 3 months during the 12-month intervention (Chalasani et al., 2018; European Association for the Study of the Liver EASL, 2021).

The potential economic implications of improvements in blood pressure and steatosis were not formally modeled during this study but are addressed in the Discussion section with reference to published literature.

#### 2.2.2 Pharmaceutical utilization

Pharmaceutical expenditure data were extracted from the regional outpatient drug dispensing system (EDOTTO) with patients’ prior consent (Regione Puglia, 2012a). Data were collected for the years 2018–2021 (pre-intervention, intervention, and post-intervention) and aggregated by ATC class. Each patient is automatically assigned a unique PILUR code (an alphanumeric identifier generated by the EDOTTO platform) that anonymizes personal data while allowing for longitudinal tracking of prescriptions. We linked the PILUR codes to the study IDs through a concordance table prepared exclusively for patients who had provided informed consent. The annual mean per-patient costs were calculated. Zero-cost years were retained to ensure consistent denominators. To improve robustness, only ATC classes with non-zero expenditures in at least two consecutive years were included in the longitudinal analyses, thereby excluding isolated, non-representative prescriptions. The annual mean per-patient costs and total expenditures were calculated, and inter-individual variability was visualized through boxplots. ATC-class-specific trends and a heatmap of the five most relevant ATC categories were reported. Drug utilization was analyzed as direct expenditures (€) since DDD was considered unsuitable for intermittent and variable-dose regimens in this cohort (Regione Puglia, 2012b; 2019).

Hence, annual pharmaceutical expenditures restricted to the five ATC classes with the highest cumulative costs were analyzed in the selected cohort (n = 27) using a mixed-effects model with year as the fixed effect and patient as the random intercept; repeated-measures ANOVA was conducted as a sensitivity analysis. Drug prices correspond to those established by the Regional Health Service (SSR) for reimbursable medicinal products (class A), and the related costs were recorded through the EDOTTO system.

#### 2.2.3 Long-term physical activity maintenance

The long-term physical activity maintenance assessment was based on patient self-report, using a specific questionnaire collected at follow-up. Only patients included in the clinical effectiveness analysis (n = 27) were considered. The binary response (“yes”/“no”) reflects ongoing engagement in regular physical activity at 3 years post-intervention, a time point associated with a significant prognostic value in MASLD cohorts (Dinno, 2020; Barata et al., 2025). The proportion of patients reporting continued activity was calculated, along with the 95% confidence intervals (95% CIs) using the Wilson method, as recommended for binary outcomes in clinical epidemiology (Paternostro et al., 2023). The relevance of sustained lifestyle changes in determining long-term outcomes in NAFLD is well-established, supporting the inclusion of this endpoint in economic and clinical evaluations (Vilar-Gomez et al., 2015; Chalasani et al., 2018).

### 2.3 Statistical analysis

Statistical analysis was also performed to characterize the phase of clinical association. Continuous variables were summarized as mean ± standard deviation (±SD)—or median and interquartile range (IQR)—and categorical variables were summarized as counts and percentages.

Preliminarily, baseline characteristics between the entire sample (N = 43) and the selected sample (N = 27) were compared using Student’s t-test or chi-square test, as appropriate. Second, within-patient changes in continuous outcomes (blood pressure and liver enzymes) were assessed using paired t-tests after verifying normality using the Shapiro–Wilk test; when the assumptions were not met, the Wilcoxon signed-rank test was applied. Hepatic steatosis grade, treated as an ordinal variable, was analyzed using the Wilcoxon signed-rank test and reported as the median (IQR). The proportion of patients who maintained physical activity was also reported.

### 2.4 Post-hoc power analysis

The underlying idea was to show that a non-significant result occurred because the power was insufficient (Crespi, 2025). We performed a post-hoc power analysis using the Wilcoxon signed-rank test on the (post–pre) empirical 1–6 grading scale. The two-sided type-I-error level was set at 0.05. A post-hoc power analysis is an estimate of the power of a test given the observed effect size and sample size. To elicit all the eligible sample size values, we investigated the performance of the post-hoc power analysis through a simulation study by varying the power values (x-axis) and achieving the corresponding sample sizes (y-axis) in relation to the observed effect size (Wilcoxon signed-rank test). However, it is worth noting that the post-hoc power analysis has been criticized, as well-argued by Hoenig and Heisey (2001). The full analysis is available in Supplementary Material S4.

All statistical analyses were performed in R (v. 4.3.3), and data management, quality checks, and table generation were additionally carried out in Microsoft Excel for Windows (build 19127.20154). Two-sided tests were used, setting p < 0.05 as statistically significant, and 95% CIs were also computed.

### 2.5 Ethical approval

This study follows a hybrid retrospective–prospective design based on the recall and reassessment of patients originally enrolled in a previous study (2018–2019). Study protocol approval was obtained from the Ethics Committee of Istituto Tumori “Giovanni Paolo II” I.R.C.C.S (protocol n. 390 of 05 July 2023). The study is registered in clinicaltrials.gov (NCT06026293). The informed consent and privacy module is provided only in the original language as per the ICH E6 r3 (Good Clinical Practices) guidelines (International Council for Harmonisation of Technical Requirements for Pharmaceuticals for Human Use ICH, 2024). All study materials, i.e., the informed consent/privacy modules and specific study questionnaire, are reported in Supplementary Material S3.

## 3 Results

### 3.1 Cohort selection and demographics

A total of 43 patients were included in the recall cohort in 2023, following participation in a structured program of physical exercise and dietary counseling for MASLD. Demographic characteristics are presented in Table 2. The mean follow-up duration in the overall cohort (N = 43) was 4.2 months (SD ±2.7; range 0–8). To ensure the robustness of the longitudinal analyses, we restricted the analytic population to patients with at least two follow-up visits other than the baseline visit (N = 27). In this sub-group, the mean follow-up duration was 6.0 months (SD: ±1.4; range: 4–8), with the median and interquartile range both at 6 months, thus reflecting a more homogeneous and sustained retention profile. This approach ensured that the effectiveness and economic analyses were based on patients with sufficient exposure to the active intervention.

**TABLE 2:** Baseline characteristics in the entire (N = 43) and selected (N = 27) cohorts at enrollment.

Characteristic	Entire cohort (N = 43)	Selected cohort (N = 27)	p-value
Age, years (mean ± SD)	60.7 ± 7.74	59.4 ± 7.95	0.512
Age range (years)	37–80	37–72	–
Male, n (%)	20 (46.5%)	13 (48.1%)	1.000
Patient retention (months)	Mean: 4.2 ± 2.7 (range 0–8); IQR: 4 (2–6)	Mean: 6.0 ± 1.4 (range 4–8); IQR: 6 (6–6)	–

Continuous variables are reported as mean ± SD (and range, where shown) or median (IQR); categorical variables are reported as n (%). p-values refer to two-sided comparisons between ITT and PP; “–” indicates not tested/not applicable.

A comparison of baseline demographic characteristics between the selected cohort (N = 27) and the entire recall sample (N = 43) demonstrated substantial homogeneity. No statistically significant differences were observed in the mean age (59.4 ± 7.95 vs. 60.7 ± 7.74 years, p = 0.512) or gender distribution (48.1% vs. 46.5% men, p = 1.000). At t0, the selected cohort showed the following clinical status: liver function tests (ALT, AST, GGT, and total and direct bilirubin) remained within reference limits, suggesting preserved hepatic functionality. Alpha-1 antitrypsin concentrations were stable across groups, further supporting the absence of significant hepatic impairment. Glucose metabolism markers, including fasting glucose, ultra-sensitive insulin, and C-peptide, showed mean values consistent with normoglycemia, although a wide dispersion of insulin values indicated inter-individual variability in insulin sensitivity. Lipid profile parameters demonstrated desirable mean concentrations, with total and LDL cholesterol values largely within the recommended ranges and HDL cholesterol levels reflecting sex-specific differences. Triglyceride exhibited greater variability, particularly in the selected cohort, but remained below the threshold for hypertriglyceridemia in the majority of subjects. Renal function, as assessed by serum creatinine, was stable and comparable between cohorts. The anemia panel, encompassing erythrocyte (RBC) counts, ferritin, and serum iron, showed values consistent with normal hematologic function.

In 2018, pharmacological data revealed a broad spectrum of treatments consistent with the multimorbid profile typical of patients affected by MASLD. The most frequent therapeutic area was cardiovascular disease, observed in approximately 68% of the cohort, which was primarily treated with angiotensin-converting enzyme (ACE) inhibitors, angiotensin II receptor blockers, beta-blockers, and calcium-channel antagonists. Dyslipidemia represented the second most prevalent comorbidity, affecting approximately 52% of patients, as indicated by the prescription for statins (C10AA) or other lipid-modifying agents (C10AX).

Metabolic disorders, notably insulin resistance, were present in approximately 34% of individuals, as indicated by the prescription for biguanides (A10BA). Endocrine dysfunction, mainly hypothyroidism, was recorded in 18% of cases, as indicated by the use of thyroid hormone replacement (H03AA). Gout and hyperuricemia, inferred from the prescription of uric acid synthesis inhibitors (M04AA), were less common, affecting approximately 10% of the cohort, while vitamin D supplementation (A11CC), indicative of osteopenia or osteoporosis, was prescribed to 12% of patients.

A smaller proportion (≈15%) received antimicrobial treatments (J01 group), reflecting intercurrent infectious diseases rather than chronic comorbidities. The overall distribution confirmed that the majority of patients presented multiple concurrent pharmacological conditions, with an average of 2.4 ± 0.9 comorbidities per subject. Cardiometabolic conditions—hypertension, dyslipidemia, and diabetes—were the predominant triad, collectively accounting for more than 80% of the therapeutic burden at enrollment, which is consistent with the expected comorbidity pattern. The relevant data are shown in Table 3.

**TABLE 3:** A) Baseline demographic and biochemical characteristics of the selected patient cohorts. Values are reported as mean ± standard deviation unless otherwise specified, and reference intervals refer to adult populations. B) List of medications recorded for each study participant, classified according to the therapeutic drug class and the corresponding Anatomical Therapeutic Chemical (ATC) code. The table summarizes the pharmacological profiles of individual patients, highlighting exposure to cardiovascular, metabolic, endocrine, and other clinically relevant drug categories. This categorization was used to characterize baseline treatment patterns and support downstream analyses of potential drug–disease and drug–biomarker associations.

Selected cohort				
Parameter	Unit	Reference interval (adults)	N	Value
Hepatic functionality				
ALT	U/L	Male patients ∼10–45; female patients ∼7–35	27	24.52 ± 10.06
AST	U/L	∼10–40	27	22.04 ± 5.89
Gamma GT	U/L	Male patients ∼8–61; female patients ∼5–36	27	19.26 ± 8.55
Total bilirubin	mg/dL	0.3–1.2	27	0.65 ± 0.27
Direct bilirubin	mg/dL	0.0–0.3	27	0.16 ± 0.04
Alpha-1 antitrypsin	mg/dL	100–200	27	163.22 ± 34.37
Glucose metabolism				
Glucose (fasting)	mg/dL	70–99	27	99.59 ± 13.86
Ultra-sensitive insulin (fasting)	µIU/mL	∼2–20	27	18.33 ± 37.29
C-peptide	ng/mL	0.5–2.0	27	2.54 ± 2.26
Lipid metabolism				
Cholesterol (total)	mg/dL	<200 (desirable)	27	194.11 ± 43.64
HDL cholesterol	mg/dL	Male patients ≥40; female patients ≥50	27	43.63 ± 11.50
LDL	mg/dL	<100 (optimal)	23	124.87 ± 35.95
Triglycerides	mg/dL	<150	27	147.63 ± 101.10
Renal functionality				
Serum creatinine	mg/dL	Male patients 0.74–1.35; female patients 0.59–1.04	27	0.80 ± 0.16
Anemia panel				
Erythrocytes (RBC)	×10^6^/µl	Male patients 4.7–6.1; female patients 4.2–5.4	27	4.85 ± 0.38
Ferritin	ng/mL	Male patients 30–400; female patients 13–150	27	108.28 ± 84.23
Iron (serum)	µg/dL	60–170	27	84.22 ± 25.91
Baseline clinical utilities				
Systolic BP	mmHg	—	27	129.26 ± 12.91
Diastolic BP	mmHg	—	27	82.59 ± 8.70
Steatosis grade	—	—	27	2.67 ± 1.71
Comorbidities (SOC)				
Cardiac disorders	​	​	3	​
Endocrine disorders	​	​	2	​
Infections and infestations	​	​	1	​
Metabolism and nutrition disorders	​	​	4	​
Vascular disorders	​	​	9	​

Patient	Drug_class	ATC
CB11	ACE inhibitors and diuretics (C09BA)	C09BA
CB11	Antiplatelet agents, excluding heparin (B01AC)	B01AC
CB30	Third-generation cephalosporins (J01DD)	J01DD
CB27	Angiotensin II antagonists and diuretics (C09DA)	C09DA
CB27	Angiotensin II antagonists, non-combined (C09CA)	C09CA
CB27	Selective beta-blockers (C07AB)	C07AB
CB27	Preparations inhibiting uric acid formation (M04AA)	M04AA
CB34	Angiotensin II antagonists and calcium-channel blockers (C09DB)	C09DB
CB34	Antiplatelet agents, excluding heparin (B01AC)	B01AC
CB34	Biguanides (A10BA)	A10BA
CB34	HMG-CoA reductase inhibitors (C10AA)	C10AA
CB41	Angiotensin II antagonists, non-combined (C09CA)	C09CA
CB35	Other lipid-modifying agents (C10AX)	C10AX
CB35	Angiotensin II antagonists, non-combined (C09CA)	C09CA
CB35	Biguanides (A10BA)	A10BA
CB35	Dihydropyridine derivatives (C08CA)	C08CA
CB35	HMG-CoA reductase inhibitors (C10AA)	C10AA
CB44	Selective beta-blockers and thiazides (C07BB)	C07BB
CB29	Alpha- and beta-adrenergic receptor blockers (C07AG)	C07AG
CB16	Thyroid hormones (H03AA)	H03AA
CB16	Vitamin D and analogs (A11CC)	A11CC
CB39	HMG-CoA reductase inhibitors (C10AA)	C10AA
CB39	Thyroid hormones (H03AA)	H03AA
CB25	ACE inhibitors and diuretics (C09BA)	C09BA
CB25	Antiplatelet agents, excluding heparin (B01AC)	B01AC
CB25	HMG-CoA reductase inhibitors (C10AA)	C10AA
CB47	Angiotensin II antagonists and diuretics (C09DA)	C09DA
CB31	Biguanides (A10BA)	A10BA
CB36	Angiotensin II antagonists, non-combined (C09CA)	C09CA
CB36	Antiplatelet agents, excluding heparin (B01AC)	B01AC
CB36	HMG-CoA reductase inhibitors (C10AA)	C10AA
CB20	Alpha-adrenergic receptor antagonists (C02CA)	C02CA
CB20	Angiotensin II antagonists and diuretics (C09DA)	C09DA
CB20	Dihydropyridine derivatives (C08CA)	C08CA

All demographic data are reported in Supplementary Material S5.

### 3.2 Cost-effectiveness evaluation

#### 3.2.1 Health-related quality of life (HRQoL)

HRQoL was quantified as the change in QALYs between baseline and the end of the intervention in the selected subgroup. This analysis aimed to quantify the impact of the integrated intervention on overall patient wellbeing, as captured by QALYs (Supplementary Material S6).

At each visit, HRQoL was measured using the SF-36 questionnaire, which was administered every 2 months. To enable the calculation of QALYs, SF-36 scores were mapped to EQ-5D utility values using a published, validated algorithm (Ara and Wailoo, 2011). Over the study period, EQ-5D utility scores showed a slight upward trend, indicating a small improvement in HRQoL. The magnitude of this increase was consistent with the modest gain observed in the main effectiveness analyses. Detailed descriptive data by timepoint are provided in Supplementary Table S6.

This approach permits the direct quantification of health gain in terms that are suitable for cost–utility analysis and aligns with current methodological standards for health technology assessment (European Network for Health Technology Assessment EUnetHTA, 2015).

To estimate QALY gains attributable to the intervention, two statistical approaches were considered, as shown in Table 3: i) a simple difference between the first and last available utility values for each patient (pre/post, “last observation carried forward”); (ii) linear mixed-effects modeling (Cunnings, 2012), which leverages the full longitudinal dataset while accommodating missing data points and random patient effects (Table 4). Of note, the mixed-effects approach was preferred for the primary analysis, as it minimizes the impact of attrition and unbalanced observation schedules, representing two common challenges in real-world, non-pharmacological intervention studies (Cunnings, 2012; Twisk, 2013). The inclusion of all available data points improves statistical power and provides an unbiased estimate of the average QALY gain by accounting for intra-patient correlation and heterogeneity.

**TABLE 4:** Summary of cost-effectiveness results for the supervised lifestyle program.

Statistics	ICER (€/QALY)	NMB @ €25,000/QALY (€)	NMB @ €30,000/QALY (€)	NMB @ €40,000/QALY (€)
N = 10,000				
Minimum	7,155	−1,150	−1,049	−896
25th percentile	14,647	−67	208	751
Median	19,547	397	761	1,485
Mean (±SD)	21,594 (9,787)	546 (818)	942 (983)	1,736 (1,317)
Base-case NMB (€)	–	585	990	1,800
75th percentile	26,246	1,001	1,489	2,465
Maximum	121,931	2,873	3,678	5,288
Probability NMB > 0	–	0,7144	0,8387	0,9500
IQR (€)	–	−66,74–1.001,10	207,95–1.488,85	750,83–2.464,60

Values are reported as incremental cost-effectiveness ratios (ICERs, €/QALY) and net monetary benefit (NMB, €) at willingness-to-pay thresholds of €25,000, €30,000, and €40,000 per QALY. The percentiles and ranges are derived from a probabilistic sensitivity analysis (N = 10,000 Monte Carlo simulations).

Table 4 shows the summary of the average SF-36 domain scores and EQ-5D index across the study period (baseline to follow-up) in the 2018 cohort. Each row represents a single patient (CBxx code), with the mean values derived from available visits. *Exercise continuation* indicates whether the participant maintained physical activity throughout the follow-up period. Higher SF-36 and EQ-5D values denote a better physical and mental health status, respectively. The table is presented in the main text as an overview of functional and quality-of-life outcomes; detailed timepoint data (t0–t8) are provided in the Supplementary Material.

Using the mixed-effects model (Cunnings, 2012) as the reference, the mean QALY gain observed in the per-protocol population was 0.081 (95% CI: 0.001–0.161). This represents a conservative estimate of the health improvement associated with participation in the program. Notably, the lower confidence interval approaches zero, supporting a true, if limited, average benefit at the population level. Comparable QALY gains have been reported in the literature for lifestyle modifications in metabolic liver disease and in broader populations with noncommunicable chronic conditions (Eriksson et al., 2010; Fernández, 2022). Importantly, the safety profile of the intervention was excellent, and no adverse events were recorded, indicating a key advantage over the majority of pharmacological therapies (Baratta et al., 2017; Chalasani et al., 2018; Harrison et al., 2020; European Association for the Study of the Liver EASL, 2021).

#### 3.2.2 Incremental cost-effectiveness ratio (ICER) and net monetary benefit (NMB)

Based on the mixed-effects model, the supervised lifestyle intervention was associated with a mean QALY gain of 0.081. With an annual program cost of €1,440 per patient, this resulted in an ICER of €17,778 per QALY gained. The program remained well below the commonly applied WTP thresholds in Italy and Europe (€25,000–30,000/QALY).

At a WTP of €25,000/QALY, the mean base-case NMB was €585 per patient, while at €30,000/QALY the mean NMB increased to €990 (Table 4). These findings confirm that the program provides positive economic value and robust cost-effectiveness under real-world conditions.

#### 3.2.3 Sensitivity analysis

##### 3.2.3.1 One-way sensitivity analysis

**FIGURE 1:**
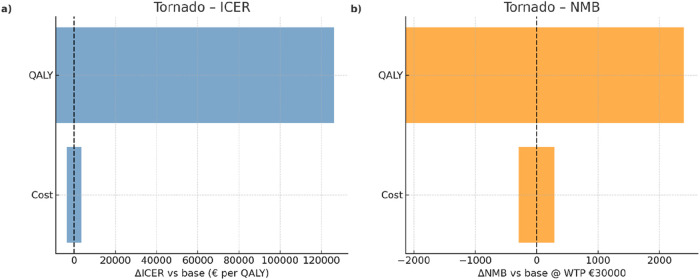
One-way sensitivity analyses for the supervised lifestyle intervention. **(a)** Tornado plot showing the variation in the incremental cost-effectiveness ratio (ICER) compared to the base case. **(b)** Tornado plot showing the variation in net monetary benefit (NMB) at a willingness-to-pay (WTP) threshold of €30,000 per QALY. QALY uncertainty had the largest influence on cost-effectiveness estimates, while intervention cost had a comparatively smaller impact.

Across the pre-specified ranges, the base-case result (∼€18,000 per QALY gained) proved robust and was far more sensitive to the QALY gain than to the program cost. Varying the QALY parameter from its lower to higher bound produced a +€135,056/QALY change in ICER relative to the baseline, whereas varying the cost parameter shifted the ICER by only +€7,111/QALY (Figure 1a). At a WTP of €30,000/QALY, the NMB remained positive throughout the tested ranges; QALY generated the widest swing (ΔNMB −€2,130 to +€2,400 vs. the base case), while cost had a modest and symmetric effect (≈±€288) (Figure 1b). Directionally, higher QALY gains lowered the ICER and increased the NMB, whereas higher costs increased the ICER and reduced the NMB. Overall, the tornado ranking consistently identified QALY as the dominant driver, with cost exerting only a minor influence. Full results for all thresholds are provided in Supplementary Table S7.

##### 3.2.3.2 Probabilistic sensitivity analysis

In probabilistic sensitivity analysis (10,000 Monte Carlo simulations; log-normal distribution for QALY gain, gamma distribution for cost, correlation coefficient ρ = 0.25), the median ICER was €19,547/QALY (interquartile range: €14,647–€26,246). All PSA results are presented in Table 4.

**FIGURE 2:**
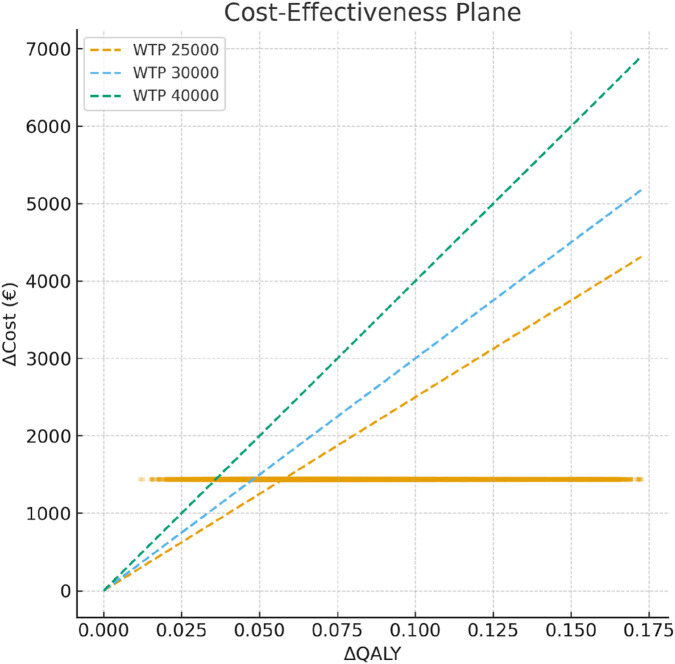
Cost-effectiveness plane based on N = 10,000 Monte Carlo simulations. Each dot represents a probabilistic simulation of incremental cost (ΔCost) and incremental effectiveness (ΔQALY). Dashed lines indicate the willingness-to-pay (WTP) thresholds of €25,000, €30,000, and €40,000 per QALY.

The cost-effectiveness plane showed that the majority of simulations fell below the €30,000/QALY isocost line (Figure 2), consistent with the CEAC profile. Scenario analyses over extended time horizons suggested further increases in expected NMB if clinical benefits persisted beyond 1 year, with robust results across tested ranges.

**FIGURE 3:**
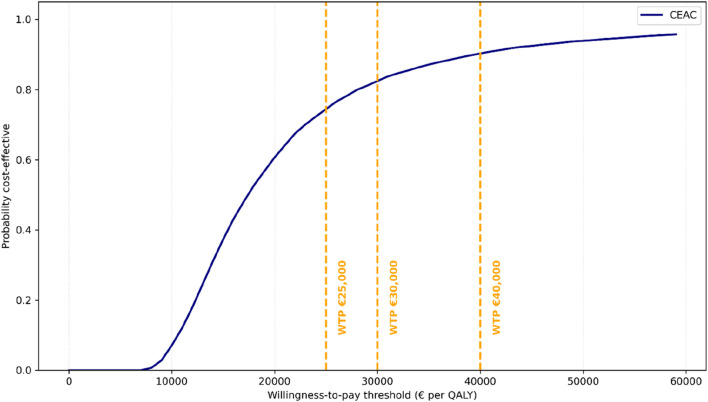
Cost-effectiveness acceptability curve (CEAC) illustrates the probability of cost-effectiveness across a range of WTP thresholds. Dashed vertical lines indicate the reference thresholds (€25,000, €30,000, and €40,000/QALY).

The cost-effectiveness acceptability curve (CEAC) is presented in Figure 3. This analysis illustrates the probability that the intervention is cost-effective compared to usual care across a range of WTP thresholds. At a WTP threshold of €25,000 per QALY, the probability of cost-effectiveness was 71%; this increased to 84% at a threshold of €30,000 per QALY and 95% at a threshold of €40,000 per QALY. Full results for all thresholds are provided in Supplementary Table S7.

#### 3.2.4 Clinical association: clinical benefits of the program

The clinical impact of the lifestyle program was evaluated across three main domains: (i) hepatic steatosis, as assessed by standardized ultrasonographic grading; (ii) blood pressure control, as compared by mean systolic and diastolic values before and after the intervention; and (iii) serum aminotransferase (AST and ALT) levels. Long-term maintenance of physical activity was also recorded as an indicator of sustained behavioral change.

Analyses were restricted to patients with ≥3 valid timepoints (including baseline), ensuring sufficient exposure to the intervention and a robust longitudinal evaluation. The results showed significant improvements in hepatic (steatosis grade) and extrahepatic markers, providing evidence of the effectiveness of structured lifestyle modifications for MASLD. Detailed statistical results are presented in the following sections.

##### 3.2.4.1 Liver steatosis

**FIGURE 4:**
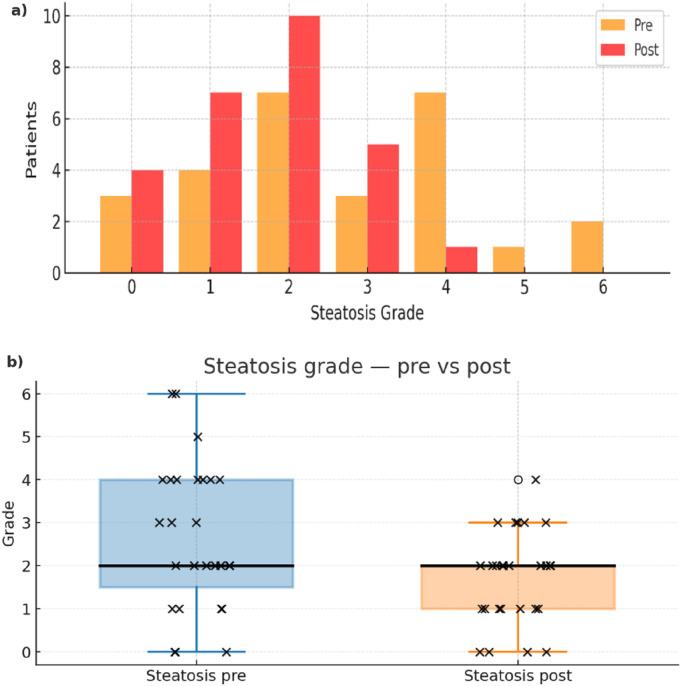
Steatosis grades before and after the intervention. Panel **(a)** depicts the distribution of patient counts across steatosis grades at baseline (pre) and after the program (post), while panel **(b)** summarizes the same data with box-and-whisker plots and overlaid individual observations (×). Relative to baseline, the post-intervention distribution shifts toward lower grades, with a lower median and fewer high-grade observations, indicating a general reduction in steatosis severity across the cohort.

Changes in hepatic steatosis grading were analyzed using the Wilcoxon signed-rank test, given the ordinal nature of the variable. At baseline, the median steatosis grade was 2 (IQR 1.5–4.0), which remained the same after the program: 2 (IQR 1.0–2.0). The median steatosis grade remained 2, but the post-intervention distribution shifted toward lower categories (IQR 1.0–2.0 vs. 1.5–4.0; Wilcoxon p = 0.007), which is consistent with a cohort-level distributional improvement rather than a confirmed per-patient minimal detectable change. The distribution shift is illustrated in Table 5 and Figure 4.

**TABLE 5:** Change in hepatic steatosis grading before and after the structured lifestyle program.

Parameter	Median pre-intervention (IQR)	Median post-intervention (IQR)	p-value
Steatosis grade	2 (1.5–4.0)	2 (1.0–2.0)	0.007

Values are presented as median and interquartile range (IQR). A significant shift toward lower steatosis grades was observed in the post-intervention distribution (p = 0.007, Wilcoxon signed-rank test).

Finally, the post-hoc analysis returned power values equal to 0.828 for the detected empirical steatosis grade delta changes by the Wilcoxon signed-rank test. Of note, a simulation study was also performed to evaluate the required sample size in relation to a set power value to compare it with the power achieved in the study (Supplementary Material S4).

##### 3.2.4.2 Blood pressure

After the structured lifestyle intervention, significant improvements were observed in both systolic and diastolic blood pressure. At baseline, the mean systolic pressure was 129.3 mmHg (SD ± 12.9), which decreased to 123.7 mmHg (SD ± 9.7) at follow-up, corresponding to a mean reduction of −5.6 mmHg (p = 0.05). Similarly, the mean diastolic pressure declined from 82.6 mmHg (SD ± 8.7) to 78.9 mmHg (SD ± 7.0), with a mean change of −3.7 mmHg (p = 0.0281) (Table 6).

**TABLE 6:** Changes in blood pressure parameters before and after the supervised lifestyle intervention.

Parameter (n = 27)	Mean pre	SD pre	Mean post	SD post	Mean delta_post–pre_	p-value
Systolic BP (mmHg)	129.26	±12.91	123.70	±9.67	−5.56	**0.05**
Diastolic BP (mmHg)	82.59	±8.70	78.89	±6.98	−3.70	**0.03**

Values are expressed as mean ± standard deviation (SD). The mean differences (Δ_post–pre_) and corresponding p-values (paired t-test) are reported. p-values < 0.05 are shown in bold.

**FIGURE 5:**
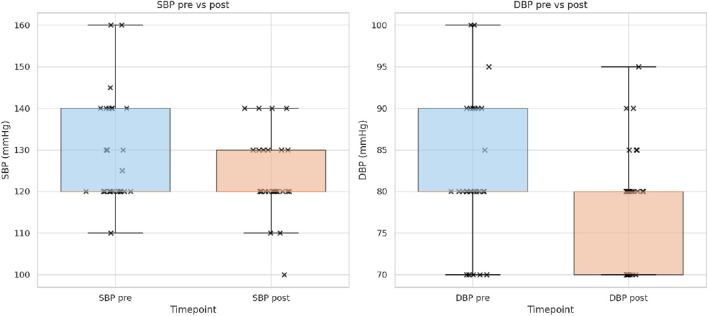
Systolic (SBP) and diastolic (DBP) blood pressure (mmHg) before and after intervention. Boxplots display the distribution of systolic and diastolic blood pressure at baseline and after the lifestyle program.

Boxplot analysis confirmed these findings, showing a visible downward shift in the median for both systolic and diastolic pressure, together with a reduction in interquartile ranges in the post-intervention group (Figure 5). The majority of individual data points were clustered below baseline values, supporting the consistency of the observed reductions across patients. Table 6 shows that significant reductions were observed for both systolic and diastolic blood pressure.

##### 3.2.4.3 Changes in serum aminotransferases (AST and ALT)

The effects of the lifestyle intervention on liver enzyme profiles were evaluated in the per-protocol cohort (N = 27) by comparing pre- and posttreatment serum levels of aspartate aminotransferase (AST) and alanine aminotransferase (ALT).

At baseline, the mean AST level was 25.3 U/L (SD ± 9.4), which decreased to 21.6 U/L (SD ± 5.1) after the intervention, with a mean change of −3.8 ± 7.0 U/L (p < 0.001). Similarly, ALT declined from 29.7 U/L (SD ± 14.4) to 23.8 U/L (SD ± 9.2), corresponding to a mean difference of −5.9 ± 9.7 U/L (p < 0.001).

**FIGURE 6:**
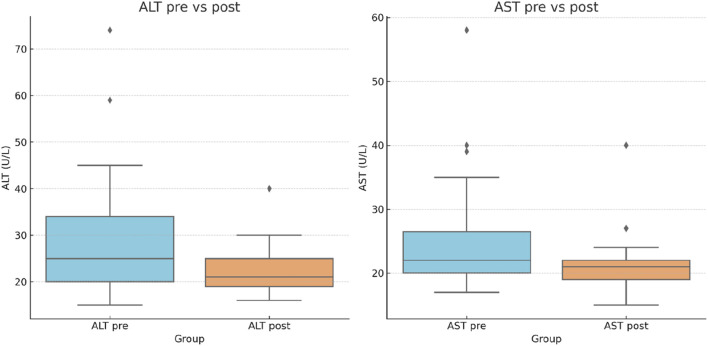
Changes in liver enzymes following the lifestyle program (N = 27). **(a)** ALT levels pre- and post-intervention. **(b)** AST levels pre- and post-intervention. Boxplots show the median, interquartile range, whiskers, and outlier values.

A significant reduction in aminotransferase levels was observed following the structured lifestyle program, as shown in Table 7 and Figure 6. The consistent decline in both AST and ALT supports the hypothesis that lifestyle modifications can attenuate hepatocellular injury in MASLD. Importantly, not only did the group averages improve, but the overall distribution of values also became narrower post-intervention, suggesting a reduced inter-individual variability and a more homogeneous improvement across the cohort.

**TABLE 7:** Serum aminotransferase (AST and ALT) levels measured before and after the structured lifestyle program in patients with MASLD.

Parameter (N = 27)	Mean pre-intervention (U/L)	Mean post-intervention (U/L)	Mean Δ (U/L)_post–pre_	p-value
AST	25.33 ± 9.39	21.56 ± 5.08	−3.78	**<0.001**
ALT	29.71 ± 14.42	23.80 ± 9.24	−5.91	**<0.001**

Data are presented as mean ± standard deviation (SD). Δ, mean change from baseline. p-values were derived from paired-sample t-tests. p-values < 0.05 are shown in bold.

##### 3.2.4.4 Changes in lifestyle behaviors

Among the 27 patients included in the analytic cohort, 15 (55.6%, 95% CI: 37.3%–72.4%) reported maintaining regular physical activity at the 2-year follow-up, whereas 12 (44.4%) did not.

This indicates that more than half of the participants were able to sustain the behavioral changes induced by the structured intervention, hypothesizing a durable impact of supervised programs on long-term lifestyle modification. However, the substantial proportion of patients who relapsed highlights the challenges of maintaining adherence outside of structured support, underscoring the importance of reinforcement strategies such as ongoing counseling, community-based exercise facilities, or digital follow-up tools.

Collectively, improvements across hepatic steatosis, blood pressure, and aminotransferase levels indicate that the intervention conferred multidomain benefits, which is consistent with a systemic effect on metabolic and cardiovascular risk factors. All raw data on clinical associations (i.e., patients’ pressure determination, AST/ALT values, and hepatic steatosis determination) are reported in Supplementary Material S8.

#### 3.2.5 Pharmaceutical expenditures

Across the 4-year observation window, total pharmaceutical expenditures decreased from €890 in 2018 to €495 in 2021 (Table 8). The mean per-patient costs followed the same trajectory, falling from €74 to €50, while the median declined from €50 to €38. Notably, the interquartile range compressed (€67 to €36), indicating not only a lower central tendency but also more homogeneous drug spending among patients over time. However, the number of patients contributing pharmaceutical expenditure data varied by year.

**TABLE 8:** Total and per-patient pharmaceutical expenditures for the analytic cohort (n = 27) by calendar year.

Year	Total expenditures (€)	Mean per patient (€)	Median (€)	IQR (€)
2018	890	74	50	67
2019	705	71	62	75
2020	643	46	25	63
2021	495	50	38	36

All values reflect conventional outpatient retail pharmacy costs only.

Analysis restricted to the five ATC classes with the highest cumulative costs (C09BB, C10AX, C02CA, N06AX, and R03AK) showed that the majority of outpatient drug expenditures were concentrated among cardiovascular and metabolic agents. Among these, ACE inhibitors and calcium antagonists (C09BB) consistently accounted for the largest share, with annual costs ranging from €308 in 2018 to €371 in 2019, and decreasing to €293 in 2021. Other relevant classes included lipid-modifying agents (C10AX), alpha-adrenergic antagonists (C02CA), antidepressants (N06AX), and adrenergics in combination with corticosteroids (R03AK), each contributing more modest amounts (typically €30–€80/year).

**FIGURE 7:**
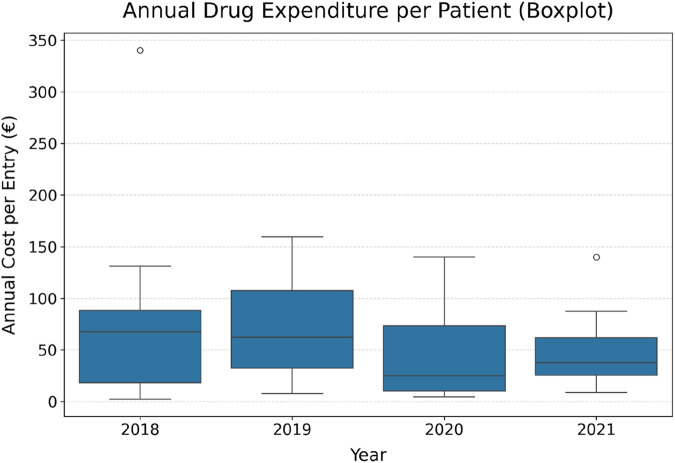
Boxplot of annual drug expenditures per patient in 2018–2021.

Boxplot analysis (Figure 7) provided additional insights, illustrating not only a progressive reduction in median expenditures but also a compression of the interquartile range, indicating greater homogeneity in spending patterns post-program. High-cost outliers became less frequent in later years, suggesting that even patients with initially elevated pharmaceutical use benefited from the program. Expenditures in the top five ATC classes showed a numerical downward trend from €125 per patient/year in 2018 to €67 in 2021. The mixed-effects model suggested a borderline significant annual decline (β = −19.2 €/year; p = 0.063), whereas repeated-measures ANOVA did not confirm a statistical significance over time (p = 0.47).

**FIGURE 8:**
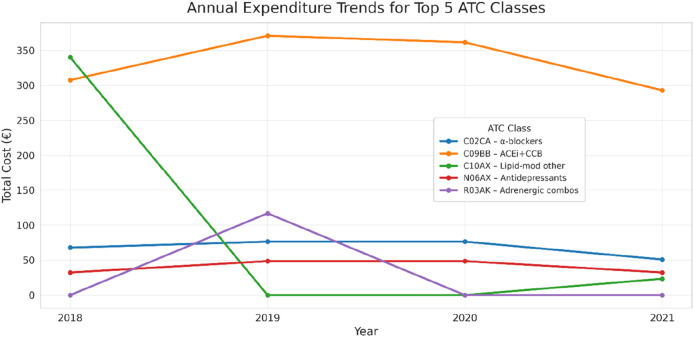
Annual expenditure trends for the top five ATC classes. Expenditure in the majority of classes declined after the intervention, while antihypertensives remained the major driver of cost.

When expenditures were disaggregated by pharmacological class, antihypertensives—particularly ACE inhibitors and calcium-channel blockers—remained the primary contributors to overall costs. In contrast, lipid-modifying agents and several other drug classes displayed sharp reductions in expenditures after the program (Figure 8).

**FIGURE 9:**
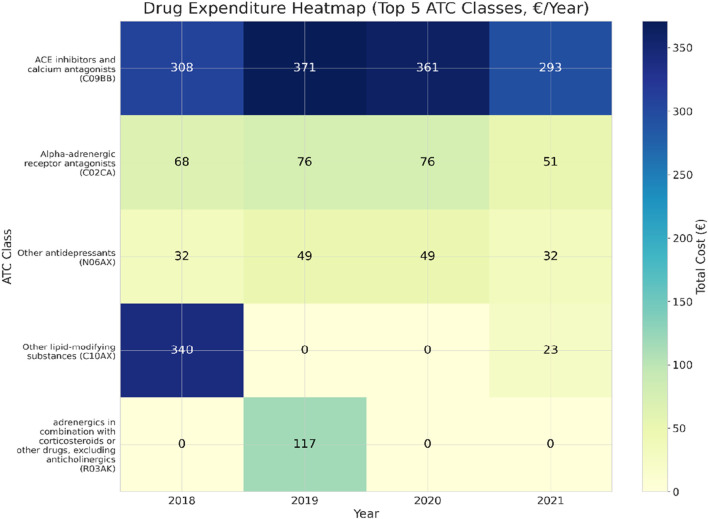
Heatmap of drug expenditures for the top five ATC classes (€/year). A general reduction in cost intensity is observed post-program.

The heatmap of the five most relevant ATC categories (Figure 9) vividly depicts this pattern, with a general attenuation of cost intensity by 2021. Trends in outpatient drug expenditures are descriptive and exploratory (mixed-effects β = −€19.2/year; p = 0.063) and may be influenced by small-n class switching and channel shifts. Apparent class-level discontinuities (e.g., C10AX and R03AK) reflect intermittent use in very few patients rather than cohort-wide de-prescribing (Supplementary Material S9).

Class-level discontinuities were driven by small-n switching and episodic use rather than broad de-prescribing. In C10AX, for example, costs fell from €340 (1 patient; 24 packages) in 2018 to €42 in 2019, were nil in 2020, and reappeared minimally in 2021 (€23). The 2018 to 2019 C10AX drop (−€298) accounted for ∼61% of the contemporaneous reduction (−€488) in total outpatient retail expenditures. R03AK was confined to 2019 (one patient, two inhalers; €117), consistent with short-term treatment of acute airway disease; it accounted for ∼3% of spending in 2019 and was absent in other years. In contrast, statins (C10AA) and RAS agents (C09) remained the predominant, stable drivers of cost across years.

The full dataset of drug dispensing, extracted using the EDOTTO regional platform and concordance table is available in Supplementary Material S9.

## 4 Discussion

This single-center pre–post study suggests that a clinically supervised lifestyle program for MASLD may be associated with measurable improvements in hepatic and extrahepatic markers alongside a favorable within-study cost per QALY. However, given the uncontrolled design and indirect utility estimation, these associative findings should be interpreted with caution. Group-level reductions in ALT and AST were statistically significant but not liver-specific and may not translate into clinically meaningful individual-level changes; therefore, we interpret them as supportive rather than definitive evidence of disease modification. HRQoL improved, with a modest yet clinically relevant QALY gain of 0.081. The use of mixed-effects modeling strengthened the analysis by accounting for attrition and within-patient correlation, supporting the robustness of the estimates. Beyond mean values, distributional analyses showed reduced variability and more homogeneous improvements, suggesting benefits for the entire cohort rather than for isolated responders. From an economic perspective, the intervention achieved an ICER of €17,778/QALY, which is well below the accepted Italian and European WTP thresholds (Russo et al., 2022). Probabilistic sensitivity analysis indicated a probability of >80% cost-effectiveness at €30,000/QALY.

Scenario analyses suggest further gains if benefits persist beyond 1 year, supporting the scalability and policy relevance of structured lifestyle programs in MASLD.

Encouragingly, more than half of the participants maintained regular physical activity at the 3-year recall, indicating the potential for durable behavioral change. This underscores the importance of reinforcement strategies—including ongoing counseling, community facilities, and digital tools—to sustain maintenance beyond structured programs.

Pharmaceutical expenditures showed a non-significant downward trend (p = 0.06), which should be interpreted cautiously as exploratory evidence rather than a robust finding. Declines were consistent across drug classes, with antihypertensives remaining the main driver of cost but lipid-modifying agents and other categories showing marked reductions. These findings highlight the broader system-level implications of lifestyle interventions beyond direct clinical outcomes (Redmon et al., 2010; Xin et al., 2019). Several pharmacological candidates, including FXR agonists, GLP-1 receptor agonists, and antifibrotic agents, are currently under investigation for MASLD/NASH; however, they remain costly and have yet to demonstrate broad long-term benefits in phase III trials (Friedman et al., 2018; Newsome et al., 2021). In contrast, supervised lifestyle interventions are immediately implementable, safe, and cost-effective, offering a pragmatic bridge until pharmacological therapies become available.

This study has several limitations that warrant consideration. The single-center design and the small analytic sample size (n = 27) inevitably limit the generalizability. Specifically, the 95% CI of the parameter estimate (i.e., mean QALY gain) obtained by the mixed-effects modeling on QALY was very large because of the small sample size. To elaborate, from a statistical point of view, poor sample sizes generate large standard errors and 95% CIs. Moreover, including only patients with ≥3 follow-up visits may have introduced attrition bias, as more adherent and motivated patients are more likely to attend repeated visits and show better outcomes. This bias may have led to an overestimation of the benefits. Long-term physical activity maintenance was based on self-report, which may overestimate adherence compared with objective measures. Health utility values were derived by mapping SF-36 scores onto EQ-5D utilities rather than through direct measurement, an approach that, while validated, is indirect and susceptible to ceiling effects. Ultrasound, while appropriate in real-world practice, has limited sensitivity and reproducibility compared with elastography or MRI-PDFF (Caussy et al., 2018). In addition, in this study, we have applied an empirical 1–6 grading scale to diagnose and follow changes in hepatic steatosis grading because no validation study has emerged in the literature to evaluate the test–retest reliability. Therefore, we could not determine whether the change of 0.96 units was relative to a meaningful change or if it was confused with the repeatability (test–retest) bias of the technology used for the assessment. It is worth noting that the changes in serum AST and ALT levels, while statistically significant, may not have direct clinical long-term significance. Accounting for this, more studies should be performed to evaluate the long-term clinical effect of this particular program of exercise. The economic analyses were focused on outpatient pharmaceutical expenditures; potential cost offsets from reduced hospitalizations or avoidance of long-term complications were not captured. The analysis of pharmaceutical expenditures was limited by the small sample size and incomplete data capture, as several patients did not provide values for specific years. Moreover, only the top five ATC classes were considered, and the observed downward trend did not reach consistent statistical significance, limiting the strength of the evidence.

Finally, the assumptions applied in the probabilistic sensitivity analysis, including the use of parametric distributions and the exclusion of extremely low QALY values, may have influenced the uncertainty estimates. Together, these factors underline the exploratory nature of our findings and highlight the need for larger, multi-center, randomized studies with longer follow-up periods and direct utility measurement to confirm and extend these results, as already done in other European countries (Balk et al., 2015; Dobson et al., 2018).

Taken together, our findings support supervised lifestyle programs as a pragmatic, cost-effective approach for MASLD, yielding measurable clinical benefits, improvements in quality of life, and reductions in pharmaceutical expenditures that result in favorable cost-effectiveness.

The integration of such interventions into the Italian NHS could address the current therapeutic gap in MASLD treatment. Structured training and certification of kinesiology professionals will be essential to ensure quality and scalability.

In the long term, these programs may contribute to healthcare savings and improved population health by i) supporting their inclusion in evidence-based national policy, ii) limiting disease progression, and iii) reducing cardiovascular and metabolic comorbidities.

At the policy level, the program may serve as a model for regional health systems, where structured lifestyle interventions could be formally tested and scaled within routine care pathways.

## Data Availability

The original contributions presented in the study are included in the article/Supplementary Material; further inquiries can be directed to the corresponding author.

## Supplementary material

Supplementary Material S1 Eligibility criteria and assessment tools. Full inclusion and exclusion criteria; protocols for anthropometry, ultrasound steatosis grading (1–6), blood pressure measurement, and laboratory panels; and SF-36 administration schedule.

Supplementary Material S2 CHEERS 2022 checklist. Completed reporting checklist for health economic evaluations (CHEERS 2022).

Supplementary Material S3 Regulatory documentation and study questionnaires (original language). Ethics-approved informed consent and privacy modules, along with the specific questionnaire used at follow-up to assess long-term maintenance of physical activity.

Supplementary Material S4 Post-hoc power simulation. Methods, R code (MASS and MKpower), simulation outputs, and Supplementary Figure S4.

Supplementary Material S5 Demographic dataset. Baseline characteristics and retention for the full recall cohort (N = 43) and the selected analysis cohort (N = 27).

Supplementary Material S6 QALY calculation dataset and models. Raw SF-36 data, mapped EQ-5D utilities, and specification/outputs of the linear mixed-effects model and the simple pre/post analysis.

Supplementary Material S7 PSA and CEAC results. Python scripts for ICER, NMB, one-way sensitivity analysis (OWSA), and PSA (CE plane, CEAC, and tornado plots), with validation routines and reproducible Excel outputs; notes on adherence to CHEERS. Full probabilistic sensitivity analysis draws and summaries; cost-effectiveness acceptability curve across €20,000–€40,000 per QALY (including CE plane coordinates and CEAC table).

Supplementary Material S8 Clinical utility raw data. Patient-level pre/post data for blood pressure, AST/ALT, and ultrasound steatosis grade (datasets underlying Supplementary Tables S5–S7 and Supplementary Figures S4–S6).

Supplementary Material S9 Pharmaceutical expenditure dataset. EDOTTO extracts (2018–2021) with annual per-patient costs, ATC class breakdowns, and the consent-based concordance (PILUR to study PATIENT_ID).

## Glossary

ALT: Alanine aminotransferase (GPT)
AST: Aspartate aminotransferase (GOT)
ATC: Anatomical Therapeutic Chemical (classification system)
BP: Blood pressure
CEAC: Cost-effectiveness acceptability curve
CHEERS: Consolidated Health Economic Evaluation Reporting Standards
CI: Confidence interval
DDD: Defined daily dose
DD: Direct distribution
DPC: Distribution on behalf (distribuzione per conto)
EQ-5D: EuroQol 5-dimension questionnaire
GGT: Gamma-glutamyltransferase
HbA1c: Hemoglobin A1c
HRQoL: Health-related quality of life
ICER: Incremental cost-effectiveness ratio
IQR: Interquartile range
ISPOR: International Society for Pharmacoeconomics and Outcomes Research
MASLD: Metabolic dysfunction-associated steatotic liver disease
MASH: Metabolic dysfunction-associated steatohepatitis
MedDRA: Medical Dictionary for Regulatory Activities
NASH: Non-alcoholic steatohepatitis (previous terminology)
NHS: National Health Service
NMB: Net monetary benefit
PSA: Probabilistic sensitivity analysis
QALY: Quality-adjusted life year
SBP: Systolic blood pressure
SD: Standard deviation
SF-36: 36-item short-form health survey
SOC: System organ classes
WTP: Willingness-to-pay
