# Supplementary material for: Economic value and clinical association of a supervised lifestyle-improving program for MASLD
Source: Front Pharmacol. 2026 Jan 16;16:1708451. doi: 10.3389/fphar.2025.1708451 (PMC12856267; doi:10.3389/fphar.2025.1708451)
Supplement: Supplementary file 1 [file DataSheet1.zip › Supplementary_materials/S3/DOMANDE FOLLOW UP.pdf]

IRCCS
Saverio de Bellis
PugliaSalute

ISTITUTO DI RICOVERO E CURA A CARATTERE SCIENTIFICO
Ente di Diritto Pubblico D.M. del 31.03.1982
Sede legale ed operativa: via Turi n.27
70013 Castellana Grotte (BARI)
C.F. – P.IVA: 00565330727

STUDIO RETROSPETTIVO E PROSPETTICO FINALIZZATO ALLA VALUTAZIONE FARMACO ECONOMICA DEL RAPPORTO COSTO/EFFICACIA DELL'ESERCIZIO FISICO SULLA PROGNOSI DELLE STEATOSI EPATICA NON ALCOLICA (NAFLD) IN PRESENZA DI PATOLOGIE CARDIO METABOLICHE CONCOMITANTI RISPETTO ALLE TERAPIE DI NORMALE PRATICA CLINICA. L'ESERCIZIO FISICO COME FARMACO

COGNOME e NOME ______________________________ CODICE______________

1 Dopo il progetto ho continuato a svolgere attività fisica

0=no	1=si per 3 mesi	2=si per 6 mesi	3= si per 1 anno	4=si > di 1 anno

2 Se si, che tipo di attività?

In palestra	
Camminata supervisionata	
Attività autonoma	

3 Quante volte a settimana?

2 volte a settimana	
3 volte a settimana	
Più di 3 volte a settimana	

4 Continui a svolgere attività?

si	

no	

5 Se hai smesso, perché?

Mancanza di tempo	
Fisicamente non ce la faccio	
Non mi piace	
Mancanza di motivazione	
Non ne ho bisogno	
Altro (specifica)	

____

____

____

6 Da quanto tempo hai smesso?

0=non ho mai smesso	1=da 3 mesi	2=da 6 mesi	3= da 1 anno	4=da + di 1 anno

DATA_____________________________ FIRMA________________
