# Supplementary material for: Economic value and clinical association of a supervised lifestyle-improving program for MASLD
Source: Front Pharmacol. 2026 Jan 16;16:1708451. doi: 10.3389/fphar.2025.1708451 (PMC12856267; doi:10.3389/fphar.2025.1708451)
Supplement: Supplementary file 1 [file DataSheet1.zip › Supplementary_materials/S3/07.QoL SF-36.pdf]

Studio retrospettivo e prospettico finalizzato alla valutazione farmaco economica del rapporto costo/efficacia dell'esercizio fisico sulla prognosi delle steatosi epatica non alcolica (NAFLD) in presenza di patologie cardio metaboliche concomitanti rispetto alle terapie di normale pratica clinica. L'esercizio fisico come farmaco

Questionario QoL SF-36

COGNOME_________________ NOME _______________________

DATA_____________________________ CODICE _____________________

QUESTIONARIO SULLO STATO DI SALUTE SF-36

Data consegna____ /____/____ N° codice paziente _____

ISTRUZIONI: Questo questionario intende valutare cosa Lei pensa della Sua salute. Le informazioni raccolte permetteranno di essere sempre aggiornati su come si sente e su come riesce a svolgere le Sue attività consuete.

Risponda a ciascuna domanda del questionario indicando la Sua risposta come mostrato di volta in volta. Se non si sente certo della risposta, effettui la scelta che comunque Le sembra migliore.

1. In generale, direbbe che la Sua salute è:

(Indichi un numero)

Eccellente	1
Molto buona	2
Buona	3
Passabile	4
Scadente	5

2. **<u>Rispetto ad un anno fa</u>**, come giudicherebbe, ora, la Sua salute in generale?

(Indichi un numero)

Decisamente migliore adesso rispetto ad un anno fa	1
Un po' migliore adesso rispetto ad un anno fa	2

Più o meno uguale rispetto ad un anno fa	3
Un po' peggiore adesso rispetto ad un anno fa..................................	4
Decisamente peggiore adesso rispetto ad un anno fa	

3. Le seguenti domande riguardano alcune attività che potrebbe svolgere nel corso di una qualsiasi giornata. La Sua salute La limita attualmente nello svolgimento di queste attività?

(Indichi per ogni domanda il numero 1, 2, o 3)

	SI, Mi limita parecchio	SI, Mi limita parzialmente	NO, non mi limita per nulla
a. Attività fisicamente impegnative, come correre, sollevare oggetti pesanti, praticare sport faticosi	1	2	3
b. Attività di moderato impegno fisico, come spostare un tavolo, usare l'aspirapolvere, giocare a bocce o fare un giretto in bicicletta	1	2	3
c. Sollevare o portare le borse della spesa	1	2	3
d. Salire qualche piano di scale	1	2	3
e. Salire un piano di scale	1	2	3
f. Piegarsi, inginocchiarsi o chinarsi	1	2	3
g. Camminare per un chilometro	1	2	3
h. Camminare per qualche centinaia eli metri	1	2	3
i. Camminare per circa cento metri	1	2	3
I. Fare i1 bagno o vestirsi da soli	1	2	3

4. **<u>Nelle ultime 4 settimane</u>**, ha riscontrato i seguenti prob1emi sul lavoro o nelle altre attività quotidiane, a causa della Sua salute fisica?

Risponda SI o NO a ciascuna domanda

(Indichi per ogni domanda il numero 1 o 2)

	SI	NO
a. Ha ridotto il tempo dedicato al lavoro o ad altre attività	1	2
b. Ha reso meno di quanto avrebbe voluto	1	2
c. Ha dovuto limitare alcuni tipi di lavoro o di altre attività	1	2
d. Ha avuto difficoltà nell'eseguire il lavoro o altre attività (ad esempio, ha fatto più fatica)	1	2

5. **<u>Nelle ultime 4 settimane</u>**, ha riscontrato i seguenti problemi sul 1avoro o nelle altre attività, a causa de1 Suo stato emotivo (qua1e i1 sentirsi depresso o ansioso)?

Risponda SI o NO a ciascuna domanda

(Indichi per ogni domanda il numero 1 o 2)

	SI	NO
a. Ha ridotto il tempo dedicato al 1avoro o ad altre attività	1	2
b. Ha reso meno di quanto avrebbe vo1uto	1	2
c. Ha avuto un calo di concentrazione sul 1avoro o in altre attività	1	2

6. **<u>Nelle ultime 4 settimane</u>**, in che misura la Sua salute fisica o i1 Suo stato emotivo hanno interferito con le normali attività socia1i con 1a famig1ia, g1i amici, i vicini di casa, i gruppi di cui fa parte?

(Indichi un numero)

Per nulla 1

Leggermente 2

Un po' 3

Molto 4

Mo1tissimo 5

7. Quanto **<u>do1ore fisico</u>** ha provato nelle ultime 4 settimane?

(Indichi un numero)

Nessuno 1

Molto lieve 2

Lieve 3

Moderato 4

Forte 5

Molto forte 6

8. **<u>Nelle ultime 4 settimane</u>**, in che misura il dolore L'ha ostacolata nel lavoro che svolge abitualmente (sia in casa sia fuori casa)?

(Indichi un numero)

Per nulla.. ….. 1
Molto poco 2
.
Un po' 3
Molto 4
Moltissimo *5*

9. Le seguenti domande si riferiscono a come si è sentito nelle ultime 4 settimane. Risponda a ciascuna domanda scegliendo la risposta che più si avvicina al Suo caso. Per quanto tempo
nelle **<u>ultime 4 settimane</u>** si è sentito...

(Indichi un numero per ogni domanda)

	Sempre	Quasi sempre	Molto tempo	Una parte del tempo	Quasi mai	Mai
a. vivace brillante?	1	2	3	4	5	6
b. molto agitato?	1	2	3	4	5	6
c. così giù di morale che niente avrebbe potuto tirarLa su?	1	2	3	4	5	6
d. calmo e sereno?	1	2	3	4	5	6
e. pieno di energia?	1	2	3	4	5	6
f. scoraggiato e triste?	1	2	3	4	5	6
g. sfinito?	1	2	3	4	5	6
h. felice?	1	2	3	4	5	6
i. stanco?	1	2	3	4	5	6

10. **<u>Nelle ultime 4 settimane</u>**, per quanto tempo la Sua salute fisica o il Suo stato emotivo hanno interferito nelle Sue attività sociali, in famiglia, con gli amici?

(Indichi un numero)

Sempre 1

Quasi sempre 2

Una parte del tempo 3

Quasi mai 4

Mai *5*

11. Scelga la risposta che meglio descrive quanto siano VERE o FALSE le seguenti affermazioni.

(Indichi un nurnero per ogni affermazione)

	Certamente vero	In gran parte vero	Non so	In gran parte falso	Certamente falso
a. Mi pare di ammalarmi un po' più facilmente degli altri	1	2	3	4	5
b. La mia salute è come quella degli altri	1	2	3	4	5
c. Mi aspetto che la mia salute andrà peggiorando	1	2	3	4	5
d. Godo di ottima salute	1	2	3	4	5
