# Supplementary material for: Economic value and clinical association of a supervised lifestyle-improving program for MASLD
Source: Front Pharmacol. 2026 Jan 16;16:1708451. doi: 10.3389/fphar.2025.1708451 (PMC12856267; doi:10.3389/fphar.2025.1708451)
Supplement: Supplementary file 1 [file DataSheet1.zip › Supplementary_materials/S3/Modulo_Privacy.pdf]

Allegato n.6

ALL. N. 6 ALLA DCS N° 96 DEL 13 MAG. 2021

Ufficio Relazioni con il Pubblico (URP)
INFORMATIVA SUL TRATTAMENTO DEI DATI PERSONALI
Articolo 13 del Regolamento Europeo 2016/679

Gentile Signora/Signore,
l'IRCCS "Istituto di Ricovero e Cura a Carattere Scientifico, "Saverio de Bellis", in qualità di TITOLARE del trattamento dei Suoi Dati Personali, (d'ora in poi, per brevità, il "TITOLARE"), La informa che i dati personali da Lei comunicati a questa struttura saranno oggetto di trattamento secondo la normativa vigente in materia di protezione dei dati personali.

OGGETTO, FINALITA' E BASE GIURIDICA DEL TRATTAMENTO

I dati personali che La riguardano, sia comuni (es.: nome, cognome, indirizzo, telefono, e-mail), sia appartenenti a categorie particolari ("dati idonei a rivelare l'origine razziale o etnica, le opinioni politiche, le convinzioni religiose o filosofiche, o l'appartenenza sindacale, nonché dati genetici, dati biometrici intesi a identificare in modo univoco una persona fisica, dati relativi alla salute o alla vita sessuale o all'orientamento sessuale della persona"), di seguito definiti congiuntamente come "dati personali", saranno trattati esclusivamente:

a) per scopi di natura amministrativa per lo svolgimento delle attività istituzionali dell'URP di questa struttura e per la gestione del reclamo/segnalazione in attuazione della normativa vigente;

b) per la realizzazione di indagini dirette a verificare il grado di soddisfazione degli utenti sui servizi offerti o richiesti, volte a migliorare la qualità dei servizi. In tal caso i Suoi dati personali saranno trattati in forma anonima ed aggregata.

c) per le attività di prenotazione e gestione di smartrecall, COD e smart ticket (effettuato attraverso l'utilizzo di software automatizzato)

Per lo svolgimento delle attività suindicate la base giuridica del trattamento risiede nell'esecuzione di un compito di interesse pubblico e sulla base di una norma di legge o di regolamento (art. 6 paragrafo 1, lett. e, GDPR), nonché, per i dati appartenenti a categorie particolari, nello svolgimento dei compiti del Servizio Sanitario Nazionale (art. 9 paragrafo 2 lettere g, h, i, j, GDPR ed art. 2 sexies, co.2, Codice Privacy).

Il conferimento dei Suoi dati personali è obbligatorio; in mancanza non sarà possibile rispondere alle Sue richieste.

MODALITA' DI TRATTAMENTO

Il trattamento dei Suoi dati personali sarà improntato al rispetto dei principi di liceità, correttezza, trasparenza, limitazione delle finalità e della conservazione, minimizzazione dei dati (i dati raccolti saranno adeguati, pertinenti e limitati a quanto strettamente necessario rispetto alle finalità per le quali sono trattati), esattezza, integrità e riservatezza.

I Suoi dati saranno trattati sia in forma cartacea, che in formato digitale e con l'adozione di misure tecniche ed organizzative per assicurare adeguati livelli di sicurezza; saranno trattati da personale dipendente o da altri soggetti che collaborano con l'Istituto, tutti debitamente a ciò autorizzati dal Titolare o da un suo delegato, nonché da soggetti appositamente designati dal Titolare quali Responsabili del trattamento dei dati personali.

PERIODO DI CONSERVAZIONE DEI DATI PERSONALI

I dati personali da Lei forniti all'IRCCS saranno conservati per il tempo necessario al perseguimento delle finalità per le quali sono trattati e successivamente nel rispetto dei termini previsti dalle disposizioni di legge e dalle vigenti procedure di scarto degli archivi documentali.

COMUNICAZIONE DEI DATI

I Suoi dati personali non verranno in alcun modo diffusi e saranno trattati nei modi previsti dalla legge e nel rispetto del segreto professionale e di ufficio.

Per le finalità sopra indicate, saranno comunicati esclusivamente ai Responsabili o agli incaricati dell'Ente per le parti del procedimento di loro competenza.

Potranno, altresì, essere comunicati ad altri soggetti di natura pubblica o privata ed agli enti competenti per finalità istituzionali e/o amministrative legate alla gestione del reclamo/segnalazione.

TRASFERIMENTO DI DATI EXTRA UE

L'IRCCS non trasferirà i Suoi dati personali verso Paesi "terzi" (non appartenenti all'UE).

PROCESSI DECISIONALI AUTOMATIZZATI

Il Trattamento dei Suoi dati personali da parte dell'IRCCS non comporta alcun processo decisionale interamente automatizzato, fatta salva l'ipotesi prevista sub c), previo rilascio di consenso su apposito modulo.

DIRITTI DELL'INTERESSATO

Nella Sua qualità di interessato al trattamento, Lei può esercitare i seguenti diritti relativamente ai Suoi dati personali, nella misura in cui è consentito dal Regolamento Europeo 2016/679:

- ☐ ottenere l'accesso ai propri dati personali ed alle informazioni relative agli stessi;
- ☐ ottenere l'aggiornamento, la rettifica dei dati inesatti o l'integrazione di quelli incompleti;
- ☐ ottenere la cancellazione dei dati personali, nei casi previsti;
- ☐ ottenere la limitazione del trattamento dei dati personali che La riguardano, nei casi previsti;
- ☐ opporsi al loro trattamento, in tutto o in parte, per motivi legittimi;
- ☐ proporre reclamo all'Autorità Garante per la Protezione dei dati personali, qualora ne ricorrano i presupposti, seguendo le procedure e le indicazioni pubblicate sul sito web dell'Autorità Garante www.garanteprivacy.it.

Il diritto alla portabilità non è esercitabile nell'esercizio di compiti di interesse pubblico quale la gestione del reclamo/segnalazione.

Per l'esercizio dei suddetti diritti, Lei dovrà presentare una istanza in forma scritta a:

TITOLARE DEL TRATTAMENTO

Istituto di Ricovero e Cura a Carattere Scientifico "Saverio de Bellis" - Ente Ospedaliero Specializzato in Gastroenterologia

Via Turi n°27 - 70013 Castellana Grotte - Bari

Email: direttoregenerale@irccsdebellis.it

RESPONSABILE DELLA PROTEZIONE DEI DATI (RPD)

Dr. Ernesto Barbone

Email: ernesto@studioconsulenzabarbone.it.

La presente informativa è consultabile sul sito internet istituzionale dell' IRCCS nell'ultima versione aggiornata.
