# Supplementary material for: Economic value and clinical association of a supervised lifestyle-improving program for MASLD
Source: Front Pharmacol. 2026 Jan 16;16:1708451. doi: 10.3389/fphar.2025.1708451 (PMC12856267; doi:10.3389/fphar.2025.1708451)
Supplement: Supplementary file 1 [file DataSheet1.zip › Supplementary_materials/S3/Ecografia_radiologia.pdf]

Istituto di Ricovero e Cura a Carattere Scientifico
"Saverio de Bellis"
Ente Ospedaliero Specializzato in Gastroenterologia
via Turi n°27 - 70013 Castellana Grotte - Bari
C.F. – P.IVA: 00565330727
Ente di diritto pubblico D.M. del 31-3-1982

Dasa-Rägister
EN ISO 9001:2008
IQ-1208-14

PROGETTO DI RICERCA ______________________

Ecografia Epatica

Esame eseguito con apparecchio Esaote MyLab70 XVG e sonda Convex da 5 MHz, a scopo di ricerca, nell'ambito del progetto di ricerca sopraindicato

Sig./ra____, nat_ il ___/____/19__ (codice _______)

Fegato:

**Dimensioni**	Normali	Aumentate	Ridotte
**Margini**	Regolari	Irregolari	
**Ecostruttura**	Omogenea normoecogena	Disomogenea	Iperecogena

Valutazione delle steatosi epatica

**Contrasto tra parenchima epatico (P. EPA) e renale (P. REN)**	**Penetrazione in profondità del fascio ultrasonoro**	**Nitidezza delle strutture vascolari, in particolare le vene**
Livello degli echi omogeneo e contrasto tra P. EPA e P. REN non evidente (0)	Parenchima epatico chiaramente visibile dalla superficie fino al diaframma (0)	Strutture vascolari chiaramente visibili (0)
Lieve discrepanza nella ecogenicità epatico-renale (1)	Presenza di opacità delle parti più profonde del fegato o mancata visualizzazione del diaframma (1)	Perdita di echi delle strutture vascolari (1)
Larga discrepanza tra echi epatici e renali (2)	Presenza di opacità delle parti più profonde del fegato e mancata visualizzazione del diaframma (2)	Strutture vascolari non chiaramente visibili (2)
**Score steatosi: □ steatosi assente (0); □ steatosi lieve (1-2); □ steatosi media (3-5); □ steatosi severa (6)**		

Colecisti:

□Alitiasica □Presenza di calcoli □Assente per exeresi
□Contratta

Note: ____
____
____
____

Castellana Grotte, ____/_____/

Dott.______________________
