# Supplementary material for: Economic value and clinical association of a supervised lifestyle-improving program for MASLD
Source: Front Pharmacol. 2026 Jan 16;16:1708451. doi: 10.3389/fphar.2025.1708451 (PMC12856267; doi:10.3389/fphar.2025.1708451)
Supplement: Supplementary file 1 [file DataSheet1.zip › Supplementary_materials/S3/MODELLO DI CONSENSO INFORMATO CER.pdf]

Unità Operativa: DIREZIONE SCIENTIFICA – UNITA' DI RICERCA CLINICA DI FASE 1
Tel. 080/4994___ - E-mail: direzionegenrale@irccsdebellis.it – PEC: dirgenerale.debellis@pec.rupar.puglia.it

MODELLO DI FOGLIO DI INFORMAZIONE AL PAZIENTE E MODULO DI RACCOLTA DEL CONSENSO PER LA PARTECIPAZIONE DEL SOGGETTO AD UNO STUDIO CLINICO

FOGLIO DI INFORMAZIONE AL PAZIENTE PER LA PARTECIPAZIONE ALLO STUDIO

- Lo studio prevede la raccolta, conservazione ed analisi di campioni biologici codificati, perciò identificabili e non-anonimi -

Studio retrospettivo e prospettico finalizzato alla valutazione farmaco economica del rapporto costo/efficacia dell'esercizio fisico sulla prognosi delle steatosi epatica non alcolica (NAFLD) in presenza di patologie cardio metaboliche concomitanti rispetto alle terapie di normale pratica clinica. L'attività fisica come farmaco

Testo proposto:

Le viene chiesto di partecipare, a fini di ricerca, ad uno studio condotto Dott. Maurizio G. Polignano della Direzione Scientifica dell'IRCCS "S. de Bellis" di Castellana Grotte (BA). Riteniamo che lei possa partecipare a questo studio perché esistono forti evidenze scientifiche che un regime alimentare corretto costituisca un valido supporto nel prevenire i rischi di malnutrizione collegate alle terapie antitumorali. Lo studio in oggetto prevede che Lei venga indirizzato, in maniera del tutto casuale, ad uno specifico trattamento alimentare. Lo studio prevede l'arruolamento complessivo di 58 pazienti già valutati, come Lei, nell'ambito delle attività clinico/assistenziali dell'Ente. Per tutti i pazienti verrà chiesto di autorizzare il gruppo di ricerca ad accedere ai dati dei sistemi informativi regionali inerenti al consumo di farmaci forniti in regime di convenzione con il SSN (non verranno valutati farmaci forniti secondo altre modalità, es farmaci di fascia C, SOP/OTC), nel periodo di riferimento. Lo studio è stato approvato dal Comitato Etico dell'IRCCS Istituto Tumori di Bari "Giovanni Paolo II".
La sua partecipazione avviene su base totalmente volontaria. Prima di decidere se partecipare o meno, lei deve leggere le informazioni che seguono e chiedere chiarimenti allo sperimentatore su qualsiasi cosa non capisca.

SCOPO DELLO STUDIO

Scopo dello studio è quantificare da un punto di vista economico il vantaggio di una terapia basata sull’esercizio fisico al fine di migliorare lo stato di salute in pazienti affetti da NAFLD eventualmente affetti anche da altra patologia cronica (ipertensione, ipercolesterolemia, insulino-resistenza, etc.). Mediante l’analisi dei dati raccolti si potrà quantificare da un punto di vista economico (minor costo per il SSN), l’effetto di questo tipo di trattamento sul suo attuale stato di salute. Questa tipologia di studio può contribuire ad orientare le politiche sanitarie verso l’adozione di modelli terapeutici efficaci e che non richiedono l’impiego di farmaci

PROCEDURE

Se decide di partecipare volontariamente a questo studio, le chiederemo unicamente di compilare i questionari denominati SAT-P e SF36, finalizzati a valutare il suo stato di salute attuale.
I dati così raccolti verranno integrati a quelli già raccolti in precedenza e rappresenteranno la base per le valutazioni oggetto di studio.

EVENTUALI RISCHI E DISAGI

Data la natura osservazionale dello studio, la partecipazione non comporta rischi e/o effetti collaterali.

BENEFICI ATTESI PER I PARTECIPANTI ALLO STUDIO

Data la natura osservazionale dello Studio, non sono previsti per Lei benefici diretti.

BENEFICI ATTESI PER LA COLLETTIVITA’

I risultati di questo studio potrebbero contribuire ad orientare le scelte in materia di programmazione Sanitaria verso l’adozione e diffusione di strategie terapeutiche basate sull’impiego di programmi di esercizio fisico . Una maggiore diffusione dell'attività fisica , raggiunta tramite un'efficace promozione, porterebbe comportare un miglioramento della condizione psicofisica della popolazione e, al tempo stesso, ridurre i costi sostenuti dal Servizio Sanitario Nazionale per trattare le patologie in gran parte causate dell'inattività fisica, stimati in circa 1,6 miliardi euro all’anno

ALTERNATIVE ALLA PARTECIPAZIONE

Lei è libero di non partecipare allo studio.

INDENNITA' PER LA PARTECIPAZIONE

La sua partecipazione allo studio non prevede alcun compenso a Suo favore.

INFORMAZIONI SUI RISULTATI DELLO STUDIO

Nella checklist alla fine del modulo di consenso, Le sarà chiesto di indicare se desidera ricevere informazioni circa i risultati di questo studio. Lei può anche scegliere di non ricevere nessuna informazione
Data la natura osservazionale dello studio e gli strumenti di calcolo che verranno impiegati non vi saranno ulteriori evidenze cliniche circa il suo attuale stato di salute. Tutte le risultanze dello Studio saranno oggetto di pubblicazione a cura del Responsabile del progetto, su riviste scientifiche internazionali.
Qualora lo desideri potrà chiedere di visionare le suddette pubblicazioni.

DIRITTO ALLA RISERVATEZZA ED ALLA PROTEZIONE DEI DATI PERSONALI

Ai sensi del Regolamento Europeo 2016/679 (GDPR) e D. Lgs. n.196 del 30 giugno 2003, "Codice in materia di protezione dei dati personali", La informiamo che i suoi dati personali saranno raccolti ed archiviati in forma cartacea e saranno utilizzati esclusivamente per scopi di ricerca scientifica.
Saranno raccolti i seguenti dati:
- I suoi dati anamnestici
- I dati realtivi al Suo piano di esercizio fisico
- I dati realtivi al Suo consumo di farmaci erogati in regime di convenzione con il SSN. Non verrano raccolte informazioni circa nessun altro farmaco da Lei acquistato al di fuori delle convenzioni (es. Farmaci a totale carico del cittadino, farmaci da banco, integratori, etc.)I

Lei ha il diritto di richiedere la situazione aggiornata dei dati registrati che la riguardano e la correzione di eventuali errori, nonché di sapere chi è il responsabile della conservazione dei dati e chi vi ha accesso. I dati raccolti saranno trattati dai ricercatori e dal personale incaricato in modo da garantire il rispetto della Sua riservatezza e potranno essere condivisi in forma codificata con altri ricercatori, sulla base del Suo consenso e nel rispetto della normativa vigente (Regolamento Europeo 2016/679 (GDPR)e D.Lgs. n. 196 del 30 giugno 2003, Codice in materia di protezione dei dati personali e successivi aggiornamenti).

Le uniche persone che saranno al corrente della Sua partecipazione allo studio sono gli sperimentatori, i medici e i ricercatori coinvolti. Nessuna delle informazioni acquisite nel corso dello studio o fornite da Lei sarà rivelata ad altri senza il Suo permesso scritto, a meno che non sia:

- necessaria al fine di proteggere i Suoi diritti o il Suo benessere (se, ad esempio, necessitasse di cure d'urgenza); *oppure*
- disposta dalla normativa sugli studi clinici.

Infatti, il personale autorizzato del Comitato Etico competente, che ha l'obbligo di verificare la corretta conduzione dello studio - potrebbe dover accedere ai documenti clinici originali dei singoli soggetti, e venire a conoscenza, quindi, del Suo nominativo, ma è vincolato dalle norme sulla riservatezza e dell'etica professionale a non rivelare ad altri la sua identità.

Al momento della pubblicazione dei risultati dello studio o della loro divulgazione in sede congressuale, non vi saranno informazioni che svelino la Sua identità, poiché i dati sono presentati in forma aggregata e, quindi, anonima.

PARTECIPAZIONE E RITIRO

La sua partecipazione al presente studio avviene su base VOLONTARIA. La mancata partecipazione non avrà alcuna conseguenza sul suo rapporto con l'IRCCS "Saverio de Bellis", o sul suo diritto a ricevere cure o altri servizi erogati da questo Ente.

Il consenso da Lei manifestato liberamente è revocabile in ogni momento, senza che ciò comporti per Lei alcuno svantaggio o pregiudizio e senza che lei debba fornire alcuna spiegazione, con il connesso diritto di richiedere che tutti i campioni precedentemente raccolti siano distrutti o resi anonimi in modo definitivo, salvo che questi non siano già stati interamente utilizzati.
Nel caso in cui lei decida di ritirare il suo consenso, nessuna nuova informazione sarà raccolta e aggiunta ai dati esistenti o alle banche dati.

IDENTIFICAZIONE DEGLI SPERIMENTATORI

Se ha domande sullo studio, contatti i seguenti nominativi

Cognome e nome	Telefono	Fax	E-mail
Maurizio Polignano	080449356		Maurizio.polignano@irccsdebellis.it
Antonella Bianco	0804994653		Antonella.bianco@irccsdebellis.it
Giuseppe Dalfino	0804994686		Giuseppe.dalfino@irccsdebellis.it
Pietro Trisolini	0804994128		Pietro.trisolini@irccsdebellis.it

DIRITTI DEI SOGGETTI PARTECIPANTI A UNO STUDIO

Lei ha il diritto di ritirare il suo consenso in qualsiasi momento, senza fornire spiegazioni e smettere di partecipare allo studio senza esserne penalizzato. Partecipando allo studio lei non rinuncia a nessuno dei suoi diritti ad assistenza e terapia incluso il risarcimento di eventuali danni derivanti dalla pratica clinica.

MODULO DI CONSENSO

FIRMA DEL SOGGETTO PARTECIPANTE ALLO STUDIO O SUO RAPPRESENTANTE LEGALE

Ho letto (o qualcuno mi ha letto) le informazioni fornite nelle pagine precedenti. Mi è stata data l'opportunità di porre domande ed ho ricevuto risposte soddisfacenti. Mi è stata inoltre consegnata una copia di questo modulo.

☐ __Desidero ricevere informazioni sui risultati dello studio, in particolare su eventuali nuovi risultati e/o possibilità diagnostiche/terapeutiche

☐ ___Non desidero ricevere alcuna informazione sui risultati dello studio

____

Nome del soggetto

____

Nome del rappresentante legale (se appropriato)

_______________________________________ ________________

Firma del soggetto o del rappresentante legale Data

FIRMA DELLO SPERIMENTATORE

Ho illustrato lo studio al/alla Sig./sig.ra
e/o al suo rappresentante legale e ho risposto a tutte le sue domande. Ritengo che abbia compreso le informazioni fornite e contenute in questo documento ed abbia espresso volontariamente il proprio consenso alla partecipazione.

____
Nome dello sperimentatore

____ _______________________________
Firma dello sperimentatore Data (deve essere la stessa della firma del soggetto)
