# Supplementary material for: Economic value and clinical association of a supervised lifestyle-improving program for MASLD
Source: Front Pharmacol. 2026 Jan 16;16:1708451. doi: 10.3389/fphar.2025.1708451 (PMC12856267; doi:10.3389/fphar.2025.1708451)
Supplement: Supplementary file 1 [file DataSheet1.zip › Supplementary_materials/S4/Post hoc power analysis.docx]

#############################

###Post hoc power analysis###

#############################

wilcox.test(dati_utility$Grado.Steatosi.Fine,dati_utility$Grado.Steatosi.Inizio,paired=T)

library(MASS)

differ<-dati_utility$Grado.Steatosi.Fine-dati_utility$Grado.Steatosi.Inizio; differ

summary(differ)

fitdistr(differ, "normal")

#install.packages("MKpower")

library(MKpower)

rxy <- function(n) rnorm(n, mean = -0.963, sd = 1.621)

sim<-sim.ssize.wilcox.test(rx = rxy, mu = 0, type = "paired", n.min = 5, n.max = 200, step.size = 1,power = 0.99,iter = 1000); sim

str(sim)

sim$n

sim$emp.power

sim$emp.power[sim$n==27] #0.828

#par(mfrow=c(2,2))

plot(sim$emp.power,sim$n, type="l", lwd=2, ylab="sample size",xlab="power",main="Power analysis for Wilcoxon test \non the steatosis grade paired samples",ylim=c(0,100))

abline(v=0.828,lwd=2,col=3)

legend("topleft",c("total sample size required","power in the study: 0.828 (n=27)"),lty=c(1,1),lwd=c(2,2),col=c(1,3),cex=0.8)


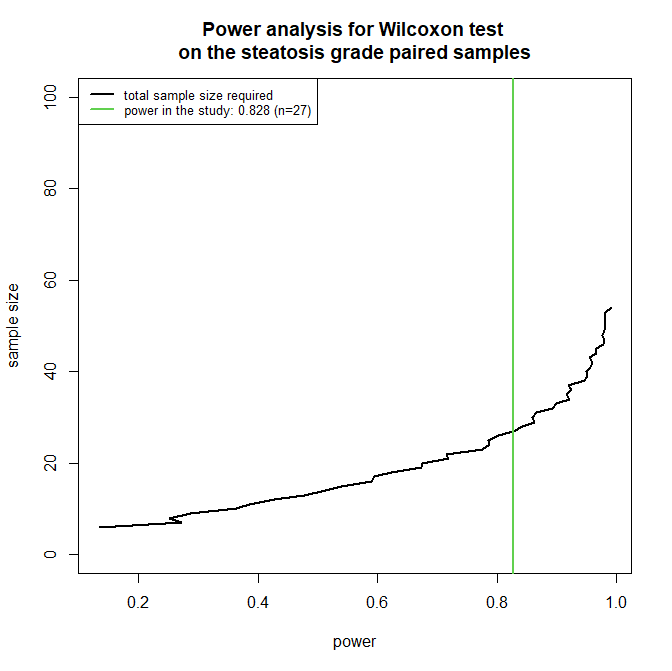


References

Crespi, C.M. Power and Sample Size in R. Chapman & Hall. 2020. ISBN 9781138591622

Hoening, J.M.; Heisey, D.M. The Abuse of Power: The Pervasive Fallacy of Power Calculations for Data Analysis. Am. Stat. 2001, 55, 19–24.

Nicole Cerabino, Martina Di Chito, Guido D., et al., Liver Fibrosis Is Positively and Independently Associated with Leptin Circulating Levels in Individuals That Are Overweight and Obese: A FibroScan-Based Cross-Sectional Study. Nutrients, 2025 Jun 1;17(11):1908. doi: 10.3390/nu17111908.

Sattin D.; Rossi Sebastiano D.; Magnani FG.; D'Incerti L.; et al. Visual fixation in disorders of consciousness: Development of predictive models to support differential diagnosis. Physiol Behav. 2021 Mar 1;230:113310. doi: 10.1016/j.physbeh.2021.113310. Epub 2021 Jan 4. PMID: 33412191.

R Core Team (2024). _R: A Language and Environment for Statistical Computing_. R Foundation for Statistical Computing, Vienna, Austria. <https://www.R-project.org/>.

Venables, W. N. & Ripley, B. D. (2002) Modern Applied Statistics with S. Fourth Edition. Springer, New York. ISBN 0-387-95457-0

Kohl M (2025). _MKpower: Power Analysis and Sample Size Calculation_. R package version 1.1, <https://github.com/stamats/MKpower>.
