# Supplementary material for: Economic value and clinical association of a supervised lifestyle-improving program for MASLD
Source: Front Pharmacol. 2026 Jan 16;16:1708451. doi: 10.3389/fphar.2025.1708451 (PMC12856267; doi:10.3389/fphar.2025.1708451)
Supplement: Supplementary file 1 [file DataSheet1.zip › Supplementary_materials/S1/S1_Original_Study_rationale.docx]

The program was conceptualized and built-up for individuals aged 18 to 65 years with a confirmed diagnosis of hepatic steatosis who were both eligible and willing to participate in a structured exercise program consisting of three weekly training sessions at a gym for 12 months. Key exclusion criteria included: (1) absolute contraindications to physical activity, as defined by the American College of Sports Medicine (ACSM) guidelines, such as acute cardiovascular diseases or prior stroke [1]; (2) significant orthopedic or neuromuscular limitations; and (3) presence of concomitant liver diseases.

For the present follow-up assessment, participants were recontacted three years after completion of the original study and asked to complete a series of questionnaires to collect updated information on their health status and lifestyle habits.

Data Collection

During the enrollment phase, participants provided written informed consent and completed a structured questionnaire collecting data on sociodemographic characteristics, medical history, and lifestyle habits. Physical activity levels were assessed using the validated International Physical Activity Questionnaire – Long Form (IPAQ-LF) [2]. Dietary intake and alcohol consumption were evaluated through the European Prospective Investigation into Cancer and Nutrition (EPIC) Food Frequency Questionnaire (FFQ) [3]. Additionally, two instruments were administered to assess health-related quality of life and daily life satisfaction: the Satisfaction Profile (SAT-P) questionnaire and the SF-36 questionnaire [4]. Blood samples and anthropometric measurements, including weight, height, and waist circumference, were collected by trained personnel following standardized protocols. Further details are available in our previous publication [5].

For the present follow-up, the SF-36 questionnaire was readministered, supplemented with additional questions focusing on potential lifestyle changes that may have occurred since the initial assessment.

Fitness Assessment Testing

To evaluate the baseline physical condition of the participants and to establish an individualized training program, three field tests were conducted to assess key components of physical fitness: cardiorespiratory endurance, muscular strength, and flexibility. These tests were repeated every month throughout the study to monitor changes in physical performance over time.

Cardiorespiratory fitness was assessed using the 2-km walk test [6], a validated measure suitable for adult populations. Muscular strength was evaluated using the push-up (or press-up) test [7], and flexibility of the lower back and hamstring muscles was assessed using the sit-and-reach test [8].

Combined Aerobic and Resistance Training

The training protocol consisted of a combined program of aerobic and resistance exercises, performed three times per week over 12 months. Each session lasted approximately 60 minutes.

Aerobic exercise intensity was determined based on maximum heart rate (HRmax), calculated using Tanaka’s formula [9]. In contrast, resistance training intensity was based on a percentage of the one-repetition maximum (1-RM) for each exercise. All participants wore heart rate monitors during training sessions to ensure continuous monitoring and control of exercise intensity.

Aerobic activities included treadmill walking, cycling, and rowing. At the same time, resistance training initially consisted of bodyweight exercises, with progression to the use of light equipment such as resistance bands, dumbbells, and bars, followed by machine-based isotonic resistance exercises.

The program began with an initial conditioning phase of 12 sessions, during which all participants performed low-intensity aerobic exercise (50–55% HRmax) and low-intensity bodyweight resistance exercises to promote gradual adaptation to physical activity.

Following this initial conditioning phase, the training protocol was structured with progressive increases in both duration and intensity to continuously stimulate physiological adaptation and improve participants' physical fitness over the long term.

Participant attendance at each training session was strictly recorded, and all participants were supervised by trained professionals during each exercise session to ensure proper technique execution and to minimize the risk of injury.

REFERENCES

1. Swain DP, Brawner CA, Medicine AC of S. ACSM’s Resource Manual for Guidelines for Exercise Testing and Prescription. Amsterdam, The Netherlands: Wolters Kluwer Health/Lippincott Williams & Wilkins; 2014.

2. Craig CL, Marshall AL, Sj"ostr"om M, Bauman AE, Booth ML, Ainsworth BE, et al. International physical activity questionnaire: 12-country reliability and validity. Medicine & Science in Sports & Exercise. 2003;35:1381–95.

3. Riboli E, Hunt KJ, Slimani N, Ferrari P, Norat T, Fahey M, et al. European Prospective Investigation into Cancer and Nutrition (EPIC): Study populations and data collection. Public Health Nutrition. 2002;5:1113–24.

4. Brazier JE, Harper R, Jones NM, O’Cathain A, Thomas KJ, Usherwood T, et al. Validating the SF-36 health survey questionnaire: New outcome measure for primary care. BMJ. 1992;305:160–4.

5. Bianco A, Franco I, Curci R, Bonfiglio C, Campanella A, Mirizzi A, et al. Diet and Exercise Exert a Differential Effect on Glucose Metabolism Markers According to the Degree of NAFLD Severity. Nutrients. 2023;15:2252.

6. Laukkanen R, Oja P, Pasanen M, Vuori I. Validity of a two kilometre walking test for estimating maximal aerobic power in overweight adults. International Journal of Obesity and Related Metabolic Disorders: Journal of the International Association for the Study of Obesity. 1992;16:263–8.

7. Canadian Society for Exercise Physiology. The Canadian Physical Activity, Fitness and Lifestyle Approach (CPAFLA): CSEP—Health and Fitness Program’s Health-Related Appraisal and Counselling Strategy. Ottawa, ON, Canada: Canadian Society for Exercise Physiology; 2003.

8. Hoeger WW, Hopkins DR. A comparison of the sit and reach and the modified sit and reach in the measurement of flexibility in women. Research Quarterly for Exercise and Sport. 1992;63:191–5.

9. Tanaka H, Monahan KD, Seals DR. Age-predicted maximal heart rate revisited. Journal of the American College of Cardiology. 2001;37:153–6.
